# Translational efficiency in gas-fermenting bacteria: Adding a new layer of regulation to gene expression in acetogens

**DOI:** 10.1016/j.isci.2023.108383

**Published:** 2023-11-02

**Authors:** Angela Re

**Affiliations:** 1 Department of Applied Science and Technology, Politecnico di Torino, 10129 Torino, Italy

**Keywords:** Biological sciences, Biochemistry, Microbiology, Biochemical research method

## Abstract

Major advances in mastering metabolism of single carbon (C_1_) gaseous feedstocks in acetogenic microorganisms are primed to fuel the transition toward environmentally sustainable and cost-efficient production schemes of biofuels and value-added biochemicals. Since acetogens grow under autotrophic energy-limited conditions, protein synthesis is expected to be controlled. This survey integrated publicly available RNA sequencing and ribosome profiling studies of several acetogens, providing data on genome-scale transcriptional and translational responses of *A. woodii*, *E. limosum*, *C. drakei*, and *C. ljungdahlii* to autotrophic and heterotrophic growth conditions. The extent of translational efficiency turned out to vary across key functional modules in acetogens’ metabolism. Translational control was confirmed to support stoichiometric protein production in multimeric complexes. Comparing the autotrophic to the heterotrophic growth condition revealed growth-dependent regulation of translational efficiency, pointing at translational buffering as a widespread phenomenon shared by acetogens.

## Graphical abstract

**Figure:**
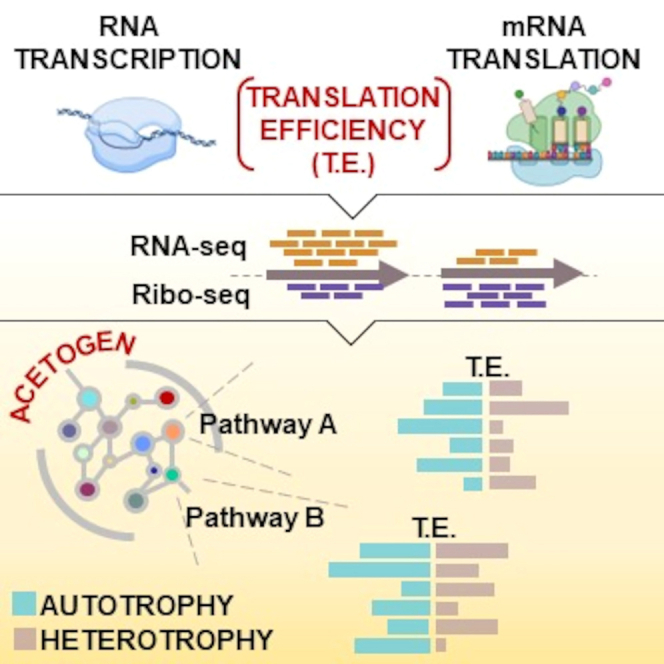

## Introduction

Handling grave global climate changes requires the rapid deployment of technological responses to accelerate the transformation to low-carbon systems. Overcoming the production systems’ inertia in committing to environmental sustainability is challenging.^1^ Microbial gas fermentation has acquired traction among the available environmental biotechnologies that support long-term mitigation goals^2^ since it can contribute to reinvent chemicals’ manufacturing using greenhouse gas emissions as feedstocks.^3^ Carbon oxides such as carbon monoxide (CO) and carbon dioxide (CO_2_) intercepted with this technology can be waste gases of the fossil-based power and the industry sector as well as gasified solid waste such as nonrecyclable municipal solid waste.^4^^,^^5^

Microbial platforms for industrial gas fermentation are acetogenic microorganisms whose distinctive metabolic trait is the usage of the Wood-Ljungdahl pathway (WLP) that carries out efficient carbon fixation coupled to energy conservation.^6^^,^^7^ From two molecules of CO_2_, by the WLP, acetogens produce acetyl-coenzyme A (CoA) and, from that, can produce acetate, often concomitantly, with versatile multi-carbon chemicals.^8^^,^^9^^,^^10^ The increasing technological maturity achieved by gas fermentation is witnessed by its recent commercial deployment.^3^^,^^11^^,^^12^^,^^13^ Notwithstanding the advances achieved by such technology, the production of long-chain or ATP-demanding molecules is challenging, due to the undoubtedly energy-limited lifestyle of acetogenic bacteria.^7^ To extensively explore the potential of gas fermentation in climate mitigation practices, several trajectories are being delineated.^14^ On one side, it is possible to combine the high substrate flexibility and product selectivity shown by acetogens with the product diversity provided by aerobes within integrated biotechnological paradigms.^4^^,^^14^ Noticeable advantages lent by this approach are the increase in achievable titers and productivities, and the widening of the portfolio of affordable bio-commodities. On the other side, synthetic biology is being actively exploited to expand the acetogens’ one-carbon valorization capabilities and build valuable chassis organisms for industrial bioprocesses.^15^

A primary objective in synthetic biology is controlling expression of genes to improve cell viability and growth^16^^,^^17^ or to evoke a certain phenotype in the acetogen of interest.^18^^,^^19^^,^^20^^,^^21^ Regardless of whether the engineered coding sequences are native or heterologous, tunable gene expression levels are desirable to programmatically act on acetogenic carbon,^22^^,^^23^ energy,^24^ electron^25^ balancing, metabolic flux distribution,^26^ substrate consumption rate,^27^^,^^28^ and regulatory mechanisms.^29^^,^^30^^,^^31^ A great deal of synthetic biology applications in acetogens leverage regulatory mechanisms of gene expression at the transcriptional layer whereas acetogenic bacteria are naturally regulated at multiple layers.^22^^,^^32^^,^^33^^,^^34^ The clear preference toward transcription-based engineering solutions runs the risk of not fully satisfying desirable features of gene expression control systems such as harmonization of gene expression with the metabolism and space constraints of the chassis under development.^35^^,^^36^ For instance, extremely abundant mRNAs potentially imply excessively high transcription and translation costs, consequently causing engineered strains to drift away from the target phenotype. Protein synthesis is by far the largest consumer of energy during cellular proliferation.^37^ Since acetogens grow at the thermodynamic edge of life,^7^ it is particularly important in acetogens that the process of mRNA translation remains in excellent synchrony with cellular metabolic needs and energy supply. Furthermore, control of translation can fulfill requirements such as minimizing phenotypic variability,^38^ or engineering templated polymer production according to genetic code expansion criteria.^39^^,^^40^

Here, we take advantage of several genome-scale transcriptome and translatome datasets publicly available to provide a fresh take on the extent to which protein synthesis is controlled in acetogens. The study gathered evidence that crucial metabolic pathways exhibit different translational efficiencies and that, under different growth conditions, translational efficiency is differentially regulated in response to resource availability. The study also provides several examples showing translational efficiency is instrumental to achieve stoichiometric protein synthesis in functionally relevant protein complexes. The overview here provided suggests a deeper understanding of translational regulation could provide actionable insights for manipulating quantitative behavior of acetogens and improving chemicals’ bioproduction by means of rational strain engineering tools.

## Genome-scale determination of transcriptional and translational expression levels

For the full realization of their biotechnological potential, acetogens demand a systematic understanding of their gene expression regulatory processes. The term “gene expression” is often used synonymously with mRNA measurements. Rather, in the gene expression flow total mRNAs and mRNAs engaged in translation generally provide non-overlapping readouts, with both useful for connecting genetic information and cellular phenotype. Until recently, precisely monitoring translation, which is the process by which a ribosome reads an mRNA template to guide protein synthesis, was far more challenging than was measuring mRNA levels by RNA sequencing (RNA-seq). The advent of the ribosome profiling approach, which was originally described by Ingolia et al.,^41^ has so far contributed to the characterization of ribosome dynamics in prokaryotes from the recruitment of the 40S to the elongation of 80S ribosomes across the open reading frame (ORF) and the recycling of post-termination ribosomes. Ribosome profiling is a deep-sequencing-based tool (Ribo-seq) that affords the detailed measurement of protein translation at sub-codon resolution globally and *in vivo.*^42^ The Ribo-seq technique exploits the fact that a translating ribosome protects from nuclease activity a discrete stretch of ∼30 nucleotides on its mRNA template. Sequencing of these ribosome-protected fragments (RPFs), termed ribosome footprints, allows us to keep track of the position of the ribosome at the time at which translation was halted. The density of protected fragments on a given transcript is deemed to provide an estimate for protein synthesis rate.^42^

Even though, to date, several acetogens have been sequenced and major genomic characteristics of model acetogens have been identified,^43^ relatively few studies are currently available that employ multi-omics approaches to portray translational profiles and decipher translational regulatory mechanisms in these microorganisms. We collected publicly available combined RNA-seq and ribosome profiling datasets that provide insights on genome-scale transcriptional and translational responses of acetogens under heterotrophic and autotrophic growth conditions.^24^^,^^44^^,^^45^^,^^46^ As detailed in the methods section, during the assembly of datasets, inclusion criteria were defined to ensure that the retrieved studies were homogeneous enough not to severely affect the validity of the following systematic overview. Dataset selection ensured that autotrophic and heterotrophic conditions were comparable across studies, and that samples for high-throughput sequencing analysis were collected in sufficient number of replicates in all studies at similar time points of growth phase. The acetogens involved in our analysis included *Eubacterium limosum* (*E. limosum*) ATCC 8486,^47^ *Acetobacterium woodii* (*A. woodii*) DSM 1030,^48^ *Clostridium ljungdahlii* (*C. ljungdahlii*) ATCC 55383,^49^ and *Clostridium drakei*^50^ (*C. drakei*). When the transcriptional levels (number of reads or fragments per kilobase of transcript per million read/fragments mapped) were compared between autotrophic and heterotrophic conditions for each microorganism and dataset, Spearman’s rank correlation (rho) varied between 0.81 (p value <2.2e^-16^) in *A. woodii* and 0.87 (p value <2.2e^-16^) in *C. drakei*. A similar tendency was observed also when comparing the translational levels (number of reads per kilobase per million RPFs) between autotrophic and heterotrophic conditions, with Spearman’s rank correlation ranging from 0.81 (p value <2.2e^-16^) to 0.86 (p value <2.2e^-16^) apart from *C. drakei* where it decreased to 0.33 (Figures S1–S4).

## Divergent differentially expressed functional gene sets at the transcriptional and translational levels

For each acetogen, genes were ranked according to the expression changes (fold change [FC] in log (base 2) scale) between the autotrophic and heterotrophic conditions at the total mRNA level and translated mRNA level, respectively. When comparing gene ranks at the two gene expression levels, Spearman’s rank correlation values were found to remarkably vary from 0.20 (p value <2.2e^-16^) in *E. limosum* and *C. drakei* to 0.72 (p value <2.2e^-16^) in *C. ljungdahlii*. I then explored how the moderate correlation between the gene expression changes at the transcriptional and translational levels reflected in biological functionalities. Functional gene set analysis was carried out to identify functional gene sets that are differentially expressed when comparing the autotrophic to the heterotrophic growth condition. The main outcome of this analysis was the poor overlap between differentially regulated functional classes identified at the transcriptional and translational levels. Secondly, few functional classes at either level were common to various acetogens here considered (Figure S5). For instance, ribosomal structural constituents were found downregulated at both levels uniquely in *E. limosum* whereas they tended to be transcriptionally upregulated in *A. woodii* and transcriptionally downregulated in *C. drakei* and *C. ljungdahlii*. A detailed discussion of differentially expressed gene sets identified uniquely at the transcriptional level or at the translational level or identified at both levels is provided in the supplemental information.

## Genome-scale determination of translational efficiency

**Figure 1:**
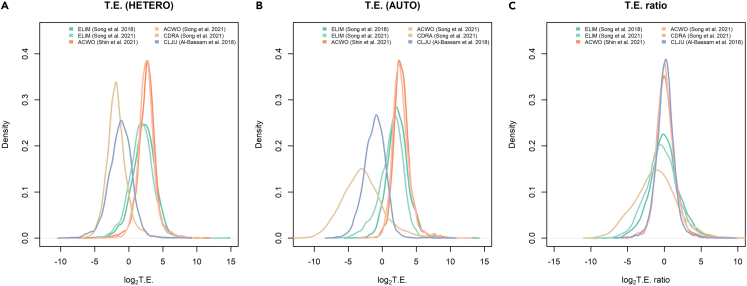
Genome-scale detection of species-specific traits for translational efficiency in acetogens (A) Density plots of translational efficiency values computed overall genes in each acetogen under heterotrophic growth condition. (B) Density plots of translational efficiency values computed overall genes in each acetogen under autotrophic growth condition. (C) Density plots of translational efficiency ratios computed, in each acetogen, between the autotrophic and heterotrophic condition. Translation efficiency and TE ratio are reported in log(base 2) scale. Figure panels display the datasets originating the total and translated mRNA levels for each acetogen.

To assess the extent of translational regulation, translational efficiency (TE) of each gene was defined as its RPF density normalized by its mRNA level.^41^ The distribution of translational efficiencies computed under each growth condition turned out to be significantly different from normal distribution in all the acetogens overviewed here (Shapiro-Wilk's normality test, significance level = 0.01). The wide range of translational efficiency values highlighted a substantial translational control of gene expression (Table 1). Noteworthy, translational efficiency distributions remarkably differ among acetogens (Kruskal-Wallis rank-sum test, p value <2.2e^-16^ under both growth conditions), with *C. drakei* and *C. ljungdahlii* showing generally lower translational efficiency values compared to *E. limosum* and *A. woodii* (Figures 1A and 1B). Recent studies delivered preliminary insights into gene features that could influence translational efficiency. More precisely, a few *cis*-acting regulatory elements of translational control were highlighted in the 5′ UTR of an mRNA. One of the key determinants of translational efficiency emerged in *C. ljungdahlii* is the ribosome binding site (RBS) motif conservation and the RBS affinity to the 3′ end of the 16S rRNA of the 30S ribosomal subunit,^24^ the so-called anti-Shine-Dalgarno sequence (aSD). Besides the SD-aSD interaction, the presence of adenosin-uracil (AU)-rich stretches upstream of the RBS is thought to regulate translation initiation by facilitating the 30S chaperone activity that intervenes in the docking and unfolding of structured mRNAs and contributes to correctly position the initiation codon inside the decoding channel.^51^ Notably, the AU content at upper RBS region was found to positively impact *C. ljungdahlii* translational efficiency in all growth conditions.^24^

**Table 1:** Variability in translational efficiency in acetogenic bacteria

Strain	Dataset	Growh	0%	25%	50%	75%	100%	Shapiro-Wilk test p value
*E. limosum*	Song et al.^46^	Hetero	−5.5	1.2	2.3	3.3	14.0	<2.2e^-16^
*E. limosum*	Song et al.^44^	Hetero	−5.9	0.7	1.8	2.9	13.6	<2.2e^-16^
*E. limosum*	Song et al.^46^	Auto	−4.9	1.3	2.2	3.2	13.5	<2.2e^-16^
*E. limosum*	Song et al.^44^	Auto	−6.4	0.4	1.6	2.5	12.3	<2.2e^-16^
*C. drakei*	Song et al.^44^	Hetero	−7.4	−2.7	−1.9	−1.0	6.1	<2.2e^-16^
*C. drakei*	Song et al.^44^	Auto	−11.9	−4.9	−3.0	−1.2	8.5	7.5e^-12^
*C. ljungdahlii*	Al-Bassam et al.^24^	Hetero	−9.6	−2.3	−1.2	−0.1	8.3	<2.2e^-16^
*C. ljungdahlii*	Al-Bassam et al.^24^	Auto	−7.5	−2.1	−1.0	−0.1	10.0	<2.2e^-16^
*A. woodii*	Shin et al.^45^	Hetero	−8.4	1.9	2.7	3.4	11.3	<2.2e^-16^
*A. woodii*	Song et al.^44^	Hetero	−4.2	1.9	2.5	3.2	10.2	<2.2e^-16^
*A. woodii*	Shin et al.^45^	Auto	−1.8	2.1	2.8	3.5	10.3	<2.2e^-16^
*A. woodii*	Song et al.^44^	Auto	−2.8	1.9	2.6	3.4	12.5	<2.2e^-16^

The table displays the values corresponding to different percentiles of the translational efficiency distribution in each acetogen. Translational efficiency values are expressed in log (base 2) scale. Shown is the p value of the Shapiro-Wilk's test for normality of the translational efficiency distribution in each acetogen under autotrophic or heterotrophic condition.

Furthermore, translational efficiency between autotrophic and heterotrophic conditions scarcely agreed, with Spearman’s rank correlation values varying from 0.20 to 0.45 except for *C. ljungdahlii* where it increased to 0.71 (Table S1). Changes in the ratio between the translated mRNA and total mRNA levels between the autotrophic and heterotrophic growth conditions (TE ratio) were then used to gauge growth-dependent translational regulation. The distribution of TE ratios recorded between the two growth conditions significantly differed from normal distribution in all the acetogens overviewed (Shapiro-Wilk's normality test, significance level = 0.01) and was indicative of substantial changes in translational regulation according to the growth condition (Table 2; Figure 1C). In particular, translational efficiency tended to increase under the autotrophic compared to the heterotrophic condition in *A. woodii* (Wilcoxon's signed rank test, p value = 1.22e^-13^ according to the dataset by Shin et al.^45^ and p value = 3.96e^-09^ according to the dataset by Song et al^44^) and *C. ljungdahlii* (Wilcoxon's signed rank test, p value = 6.20e^-11^) whereas the opposite behavior occurred in *C. drakei* (Wilcoxon's signed rank test, p value <2.2e^-16^). The decrease in translational efficiency was observed also in *E. limosum* but was not confirmed in both datasets (Wilcoxon's signed rank test, p value <2.2e^-16^ according to the dataset by Song et al.^44^). Therefore, acetogens seemingly adopt different translational regulatory strategies in response to the change from heterotrophic to autotrophic growth condition (Figure S6). *C. drakei* and *E. limosum* tend to reduce translational efficiency as a general strategy to cope with a condition of insufficient energy. Instead, *A. woodii* and *C. ljungdahlii* seem to execute selective increase of translational efficiency of individual mRNAs within the plausible reallocation of protein synthesis capacity in response to limited resources under autotrophic condition. In past years, some gene features have been suggested to be relevant in relation to the growth-dependent regulation of translational efficiency. For translation of transcripts into proteins to take place, ribosomes need to associate with the 5′ UTR of the mRNA and to initiate translation of the ORF from the start codon. Recently, the acquisition of ribosome occupancy data in *A. woodii* 5′ UTRs led to the discovery that the RPF/RNA ratio in the 5′ UTR in comparison to the ORF region significantly increases under autotrophic growth condition when compared to the heterotrophic one (>2.8-fold; Wilcoxon's matched-pairs signed rank test, p value <0.0001).^45^ It is possible to hypothesize that the pronounced accumulation of ribosomes in 5′ UTRs of mRNAs, particularly in correspondence to the SD sequence, under autotrophic growth condition indicates ribosome inactivation and thus impediment of translational initiation, which is one of the most influential steps in translational efficiency.^52^^,^^53^ Another gene feature mildly associated with changes in translational efficiency upon growth condition is the structural accessibility of the 5′ UTRs of mRNAs to translation factors. Two studies, respectively in *A. woodii*^45^ and in *E. limosum*,^46^ showed that highly structured 5′ UTRs characterize mRNAs that decrease their translational efficiency in the autotrophic condition compared to the heterotrophic one. However, these studies base their mRNA secondary structure predictions on *in silic*o folding energy models. Therefore, assessing the effect of mRNA secondary structure on translational efficiency regulation would benefit from further investigation and possibly *in vivo* biochemical approaches such as SHAPE-MaP^54^ and DMS-MaPseq.^55^

**Table 2:** Variability in translational efficiency ratio in acetogenic bacteria

Acetogen	Dataset	0%	25%	50%	75%	100%	Shapiro-Wilk test p value
*A. woodii*	Shin et al.^46^	−4.6	−0.6	0.1	0.9	10.6	<2.2e^-16^
*A. woodii*	Song et al.^44^	−5.3	−0.6	0.1	0.8	8.7	<2.2e^-16^
*E. limosum*	Song et al.^46^	−9.6	−1.2	0.0	1.2	10.6	2.5e^-13^
*E. limosum*	Song et al.^44^	−8.2	−1.7	−0.4	0.9	8.6	7.7e^-05^
*C. ljungdahlii*	Al-Bassam et al.^24^	−6.1	−0.6	0.1	0.8	9.2	<2.2e^-16^
*C. drakei*	Song et al.^46^	−9.4	−3.1	−1.2	0.6	10.1	2.5e^-05^

The table displays the values corresponding to different percentiles of the distribution of translation efficiency ratio between the autotrophic and heterotrophic conditions in each acetogen. Translational efficiency ratios are expressed in log (base 2) scale. Shown is the p value of the Shapiro-Wilk test for normality of the distribution of the translational efficiency ratio in each acetogen.

**Figure 2:**
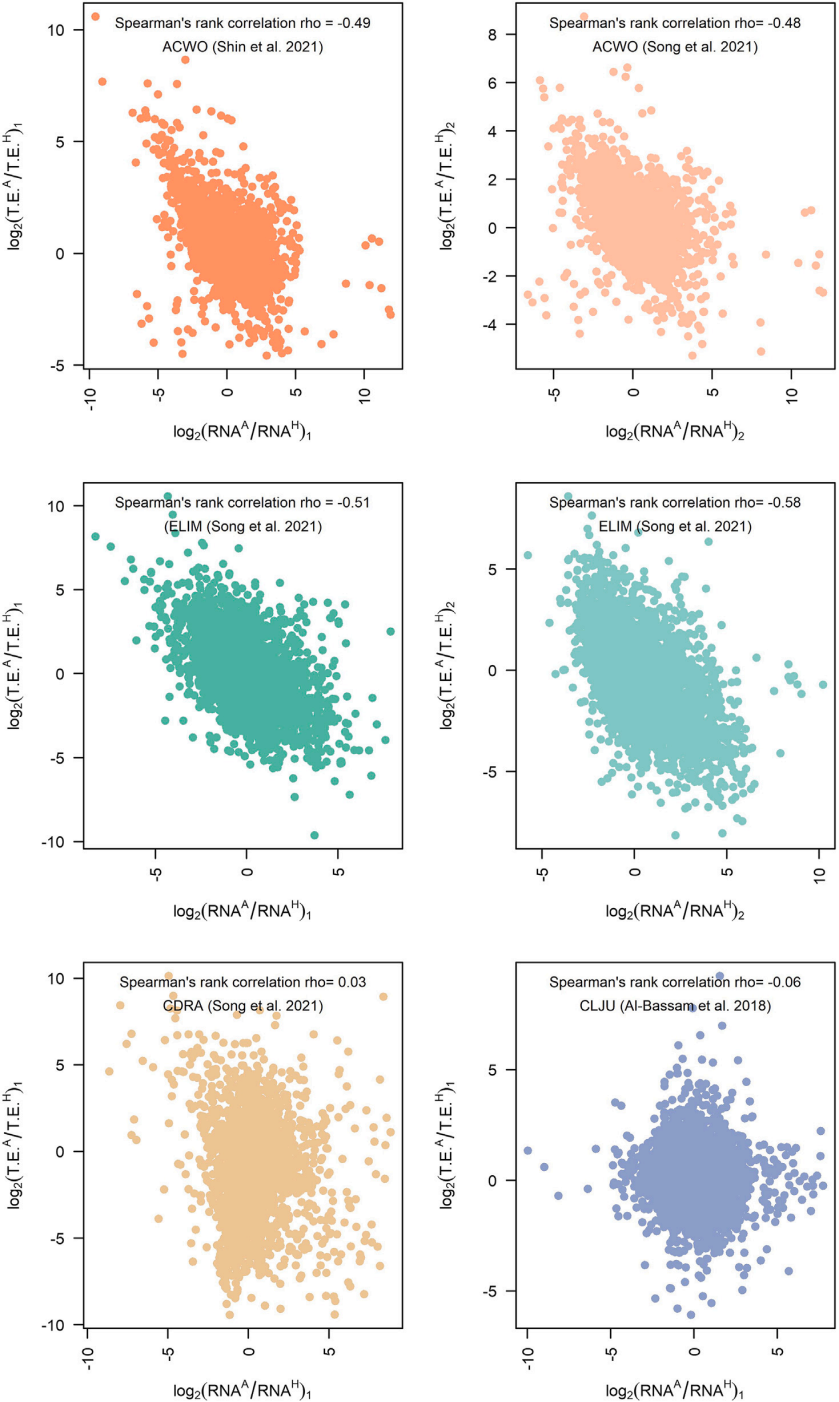
RNA sequencing and ribosome profiling reveal translational buffering in acetogens Separate figure panels plot transcriptional fold changes versus translational efficiency changes between growth conditions (autotrophic/heterotrophic) for each acetogen. Data are reported in log(base 2) scale. Each panel displays Spearman’s rank correlation for each acetogen and, if necessary, for each dataset.

**Figure 3:**
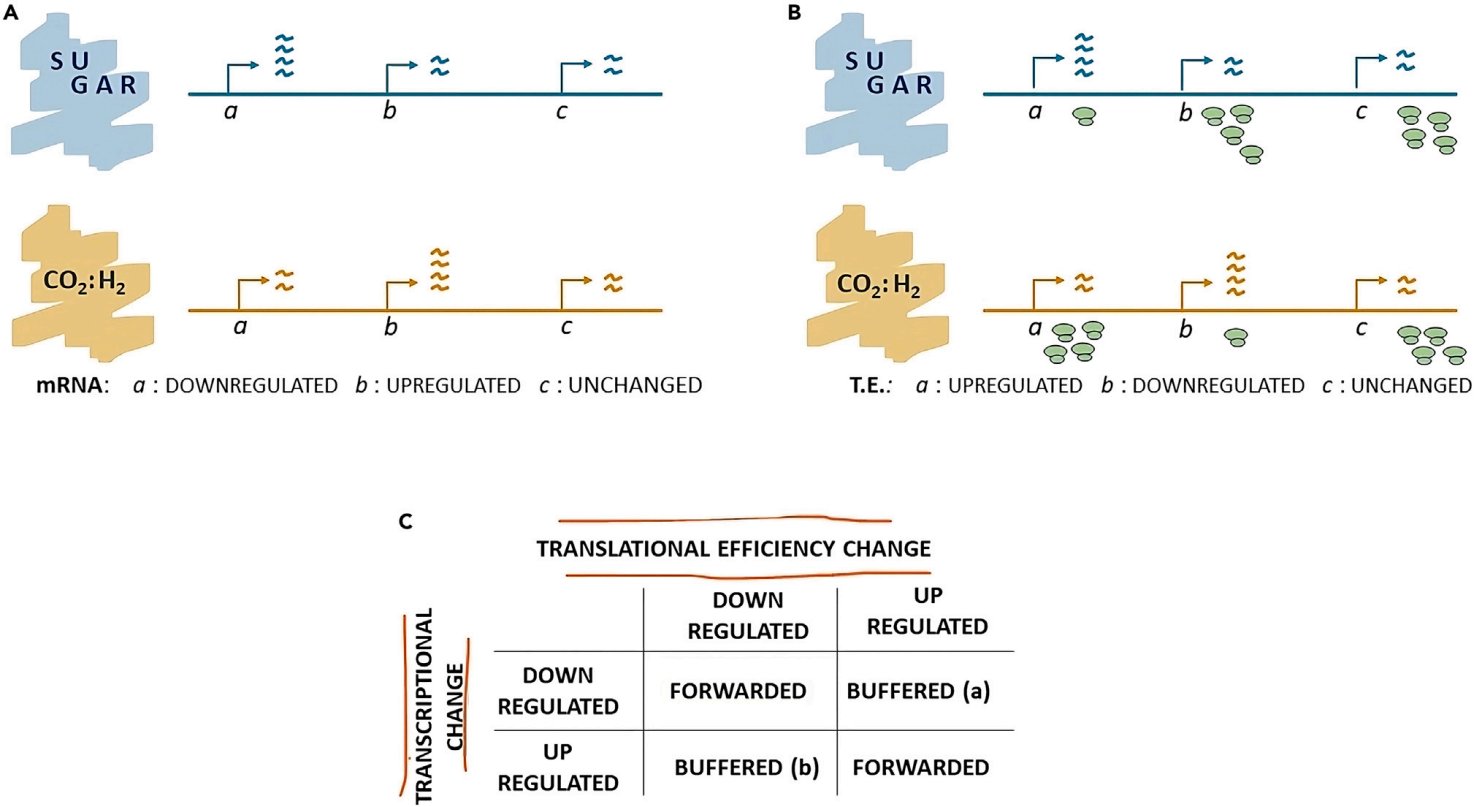
Schematic representation of translational buffering The figure exemplifies the behavior of translationally buffered genes based on fold changes of mRNA and translational efficiency between the autotrophic and heterotrophic growth condition. (A) Schematic representation of changes in mRNA abundance between the two growth conditions. (B) Schematic representation of changes in translational efficiency between the two growth conditions. mRNA counts are displayed in the upper panel while ribosomes-protected fragments are displayed in the lower panel for each growth condition. (C) If a gene is transcriptionally regulated and shows differential translational efficiency, its classification is determined based on a combination of the relative direction of change between transcription and translation efficiency. In a translationally buffered gene, the change in translational efficiency counteracts the change in mRNA abundance.

**Figure 4:**
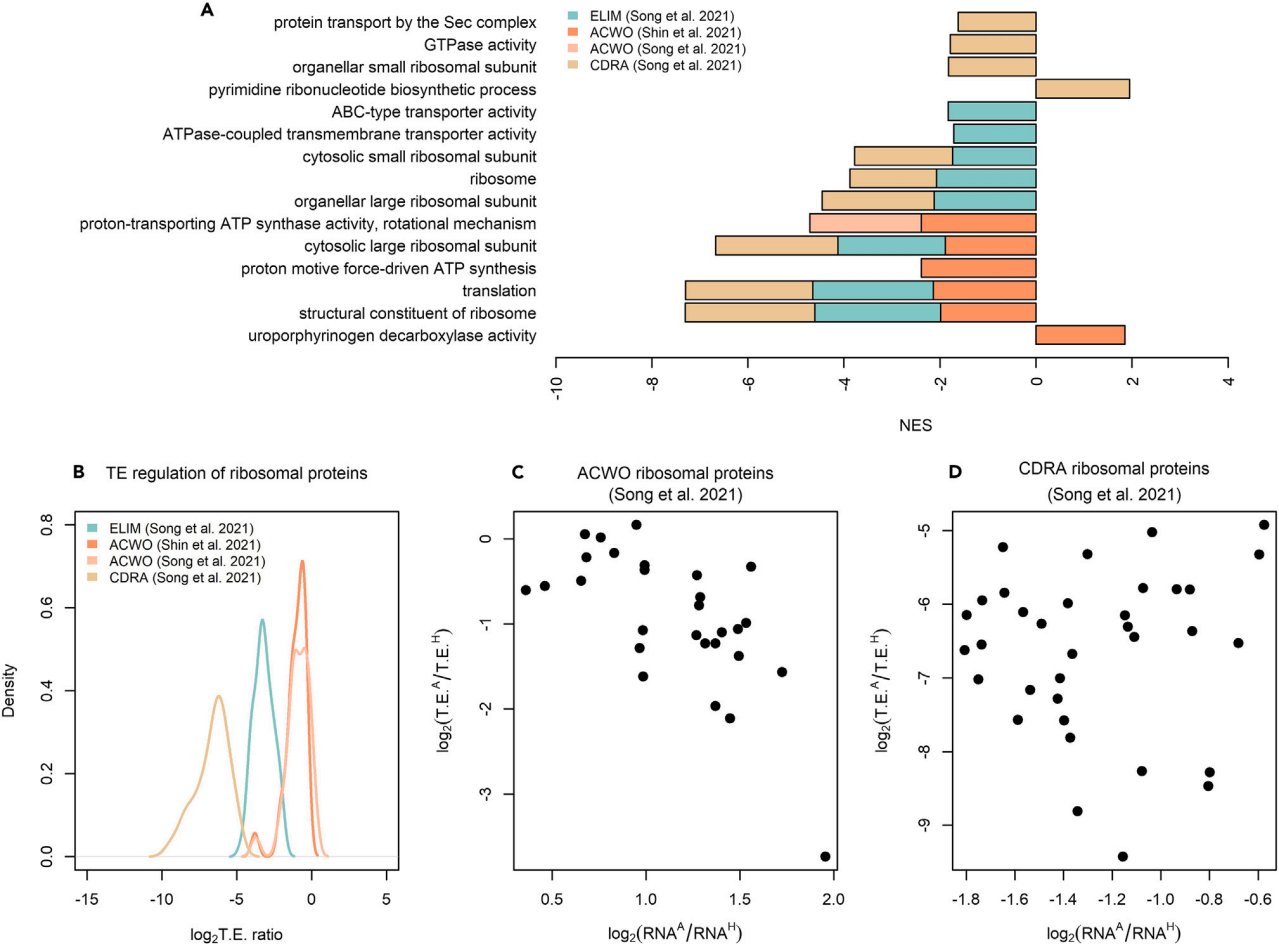
Functional gene set enrichment analysis reveals translational buffering of the ribosomal machinery (A) Barplot showing the normalized enrichment score (NES) values corresponding to differentially regulated Gene Ontology (GO) categories. Positive (negative) NES values indicate that the genes belonging to a GO category are overrepresented at the top (bottom) of the genes ranked by the ratio of translational efficiency between the autotrophic and heterotrophic growth conditions. (B) Density plots of the translational efficiency corresponding to ribosomal proteins in each acetogen. Values are reported in log(base 2) scale. (C) Relationship between transcriptional fold changes and translational efficiency change, reported in log(base 2) scale, between the autotrophic and heterotrophic conditions for *A. woodii* ribosomal proteins. (D) Relationship between transcriptional fold changes and translational efficiency change, reported in log(base 2) scale, between the autotrophic and heterotrophic conditions for *C. drakei* ribosomal proteins.

Furthermore, comparing mRNA changes and translational efficiency changes revealed a negative correlation in *A. woodii* (rho = −0.48, p value <2.2e^-16^ and rho = −0.49, p value <2.2e^-16^ according to the dataset considered), in *E. limosum* (rho = −0.51, p value <2.2e^-16^ and rho = −0.58, p value <2.2 e^-^^16^ according to the dataset considered), and to a lesser extent in *C. ljungdahlii* (rho = −0.06, p value = 0.0005) which pointed at the widespread presence of translational buffering^45^^,^^56^ (Figure 2). Translationally buffered genes feature a change in translational efficiency that counteracts the change in total mRNA (Figure 3), thus buffering the effect of transcriptional regulation.^57^ Translational buffering could, in principle, be attributed to gene-specific regulatory *cis*- and *trans*-acting factors^53^^,^^58^^,^^59^ as well as to limited protein synthesis capacity of the ribosomal machinery.^57^ Indeed, ribosome production and usage are known to differ across nutrient conditions.^60^ Gene-specific and global mechanisms are not mutually exclusive and both possible. The latter scenario appears consistent with the outcomes of the analysis carried out to dissect translational efficiency regulation between the autotrophic and heterotrophic growth condition by functional categories. Fast gene set enrichment analysis (FGSEA, q-value <0.05) was applied to the genes previously ranked according to the computed TE ratios. Intriguingly, ribosomal proteins were found to be subject to translational efficiency downregulation (*E. limosum*: adjusted p value = 1.22e^-10^; *A. woodii*: adjusted p value = 0.00082; *C. drakei* adjusted p value = 2.51e^-18^). As a matter of fact, the decrease in translation efficiency of ribosomal proteins is the most shared feature among the acetogens included in our survey, albeit at different extent across *A. woodii*, *C. drakei*, and *E. limosum* (Figures 4A and 4B). Furthermore, it is likely that the regulatory mechanisms that underpin the observed decrease of translation efficiency of ribosomal proteins could vary among acetogens. For instance, *A. woodii* genes encoding ribosomal proteins were translationally buffered since the change in translational efficiency opposes the increase in total mRNA levels under the autotrophic compared to the heterotrophic condition, as shown in Figure 4C, whereas in *C. drakei* the changes in translation rates strengthen the changes observed in mRNA abundance, as shown in Figure 4D. Hitherto, the molecular mechanisms possibly underpinning the unveiled translational buffering are a considerably underexplored territory in acetogens, and deserve future investigations.

## Pathway-specific translational efficiency changes in association with growth condition

Comparison of translation efficiency between the autotrophic and heterotrophic growth conditions was then carried out specifically for individual major pathways in acetogens’ metabolism. In addition to the WLP, which serves as an electron-accepting, energy-conserving pathway, and as a pathway for carbon assimilation, the following pathway analysis accounted for genes related to energy and redox homeostasis, glycolysis/gluconeogenesis, the pentose phosphate pathway, the incomplete reductive tricarboxylic acid cycle, by-products synthetic pathways, and multiple substrates’ utilization pathways. To this aim, genomic features of each acetogen were explored to identify the genes involved in each pathway (supplemental data item). Translational efficiency was then tested for differential regulation under the autotrophic compared to the heterotrophic growth condition for each pathway by nonparametric tests detailed in the methodological section (statistical significance = 0.01). The following discussion will limit to major pathways in acetogens’ physiology that revealed evidence of regulated translational efficiency. It is usually useful to supplement this discussion with the indication of gene expression changes at the total mRNA and/or translated mRNA levels.

### WLP

**Figure 5:**
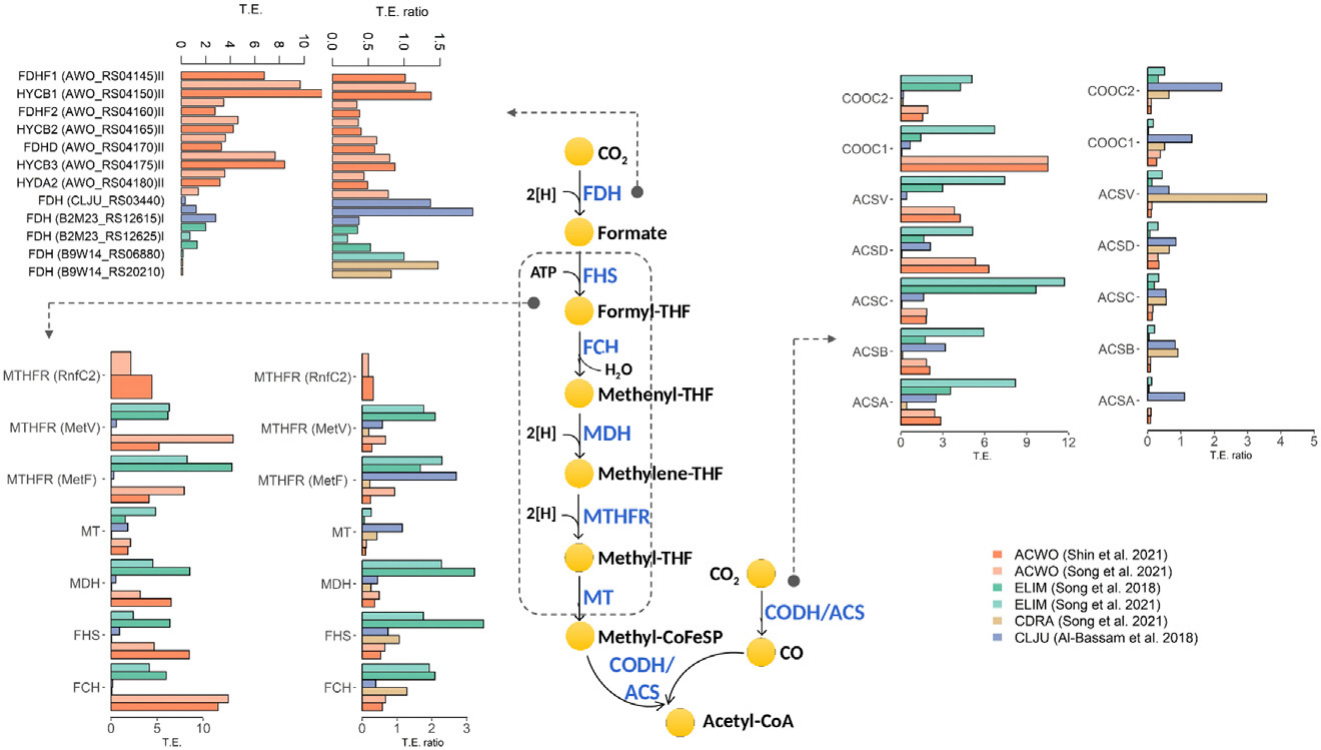
Translational efficiency and translational efficiency ratio of the Wood-Ljungdahl pathway Shown are the metabolic reactions involved in the WLP methyl and carbonyl branches. For each enzyme, the figure displays translational efficiency in the autotrophic condition and the ratio of translational efficiency between the autotrophic and heterotrophic conditions in each acetogen (color-coded as per the legend).

During autotrophic growth, one function of the WLP is CO_2_ fixation for the formation of biomass. During heterotrophic growth, this function is instead dispensable and the WLP function is to achieve a balanced redox stoichiometry by re-oxidizing reduced electron carriers generated by the oxidation of an energy-rich substrate such as glucose or fructose. The WLP is mainly composed of two linear converging metabolic branches: a carbonyl and a methyl branch (Figure 5).

### Carbonyl branch

**Figure 6:**
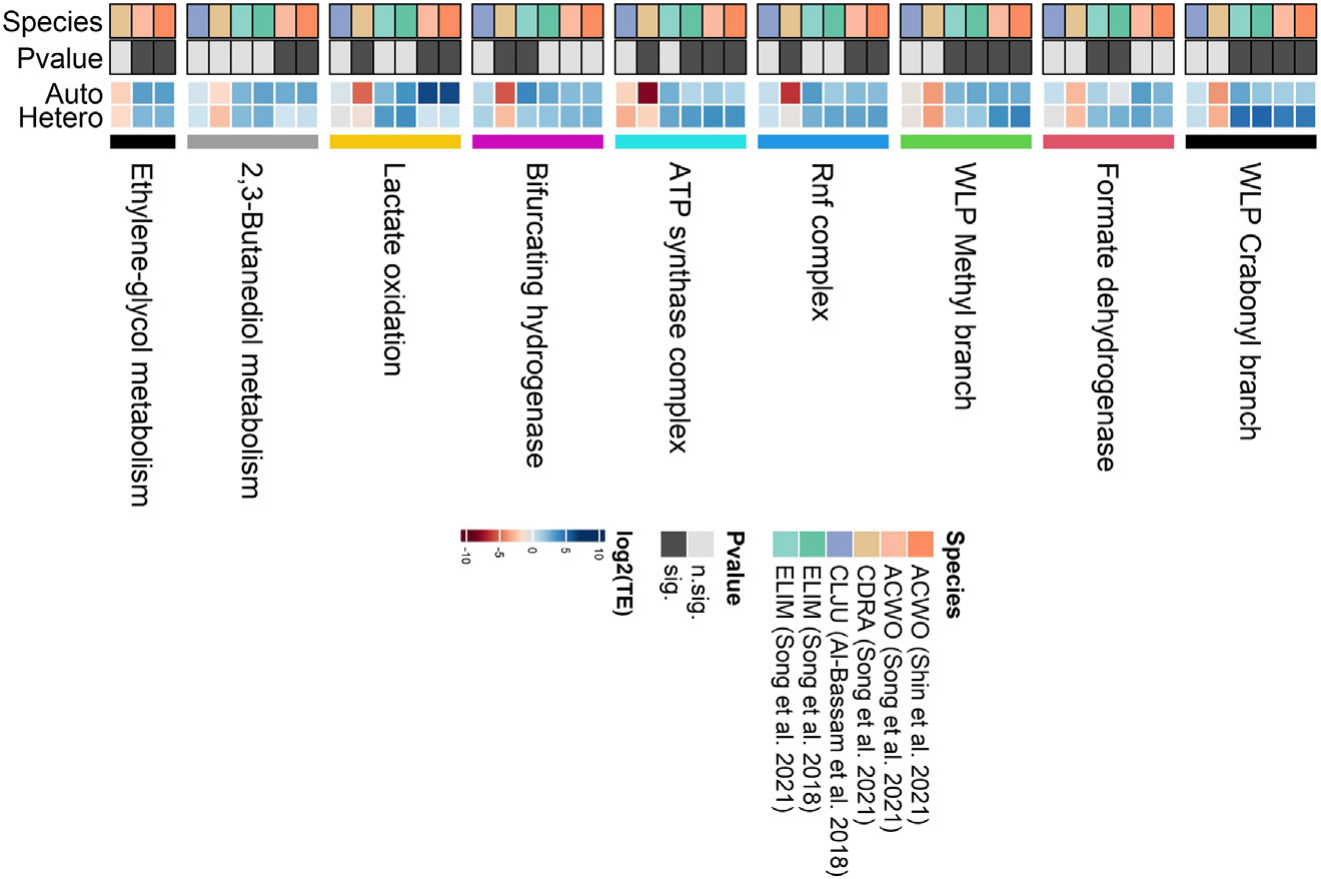
Differential translational efficiency on single-pathway basis Heatmap showing the median translation efficiency of genes belonging to pathways whose translational efficiency was assessed differentially regulated in a statistically significant way in at least an acetogen. Column annotations display whether the difference in translational efficiency between the autotrophic and heterotrophic growth conditions is statistically significant.

The carbonyl branch consists of a bifunctional enzyme, called acetyl-coenzyme A (CoA) synthase/carbon monoxide dehydrogenase (ACS/CODH). CODH catalyzes CO_2_ reversible reduction to CO, and ACS catalyzes C–C bond formation using CODH-generated CO and a methyl group to generate the key metabolic intermediate acetyl-CoA. The methyl group is produced from the WLP methyl branch and carried by an intermediate cobalamin-containing Iron-Sulfur (Fe-S) protein.^61^ When comparing the mRNA abundance with the rate of translation as estimated by the ribosome footprint density, acetogens grown heterotrophically were found to differ from each other. Indeed, in *A. woodii* and *E. limosum*, translated mRNA levels increased relative to the total mRNA levels as reflection of high translation efficiency. However, the opposite behavior occurred in *C. drakei* where translated mRNA levels were dampened with respect to total mRNA levels. When grown autotrophically, the rates of translation tended to reflect more closely the mRNA abundances owing to the statistically significant decrease in translational efficiency in most acetogens (Figure 6). *C. ljungdahlii* genes featured modest translation efficiency in either growth condition (Table 3; Figure 5). Inspecting the behavior of specific complexes suggests that translation efficiency could contribute to achieve the stoichiometric ratios that are required among the subunits for proper complex functionality. Examples are represented by the heterotetrametric ACS/CODH complex and by the methyltransferase/corrinoid Iron-Sulfur protein (MET/CoFeSP) via which a methyltransferase transfers the methyl group from methyl-tetrahydrofolate (THF) derived from the WLP methyl branch to CODH/ACS. For instance, in *A. woodii*, *acsA*, the active site of the CODH subunit for reversible oxidation of CO to CO_2_, and *acsB*, the active site of the ACS subunit, were translated in the same stoichiometric ratio (RPF_*acsA*_/RPF_*acsB*_ = 1.11 under heterotrophic condition and RPF_*acsA*_/RPF_*acsB*_ = 1.14 under autotrophic condition). The required stoichiometric ratio AcsA:AcsB (2:2) was achieved thanks to different translational efficiencies shown by *acsA* (TE_*acsA*_ = 2.07) and *acsB* (TE_*acsB*_ = 2.85). Moreover, if we remind that the MET protein, encoded by *acsE*, interacts with two COFeSPs, each of which consists of a small and large subunit encoded, respectively, by *acsC* and *acsD*, ribosomal data suggest that MET/CoFeSP rely on translational control to achieve the stoichiometric ratios of the subunits in *C. ljungdahlii* and *A. woodii*. In *C. ljungdahlii,* for instance, in autotrophic condition, *acsC* and *acsD* showed highly similar translation levels (RPF_*acsD*_/RPF_*acsC*_ = 1.00) despite different transcript levels (mRNA_*acsD*_/mRNA_*acsC*_ = 0.77) thanks to the higher translational efficiency of *acsD* with respect to *acsC* (TE_*acsD*_ = 2.11; TE_*acsC*_ = 1.63). Moreover, the translation rate of *acsE* is around 2-fold the translation rate of *acsC* and *acsD*, as expected (e.g., RPF_*acsE*_/RPF_*acsC*_ = 2.00; RPF_*acsE*_/RPF_*acsD*_ = 1.50 in *A. woodii* grown under heterotrophic condition).

**Table 3:** Quantification of mRNA counts and ribosome-protected mRNA fragments (RPFs) in selected pathways

Acetogen	Dataset	MDN(log_2_(RNA))		MAD(log_2_(RNA))		Mdn(log_2_(RPF))		MDN(log_2_(RPF))		RNA vs. RPF (p value)	
		AUTO	HETERO	AUTO	HETERO	AUTO	HETERO	AUTO	HETERO	AUTO	HETERO
WLP carbonyl branch											

*A. woodii*	Shin et al.^45^	12.42	10.38	2.31	1.37	14.19	14.81	1.54	1.28	0.03	5.41e^-06^
*A. woodii*	Song et al.^44^	11.98	10.07	2.43	1.60	13.80	14.46	1.53	1.46	0.04	2.17e^-05^
*E. limosum*	Song et al.^46^	13.59	9.48	1.13	0.58	14.51	14.15	2.13	1.61	0.04	2.06e^-05^
*E. limosum*	Song et al.^44^	12.15	9.90	1.19	0.59	14.51	14.16	2.13	1.62	3.90e^-03^	2.42e^-04^
*C. drakei*	Song et al.^44^	14.66	12.36	1.08	1.03	10.72	9.53	2.09	0.90	0.03	2.30e^-03^
*C. ljungdahlii*	Al-Bassam et al.^24^	11.16	11.32	2.64	1.67	10.97	10.20	3.50	4.28	0.51	0.62

Formate dehydrogenase											
*A. woodii*	Shin et al.^45^	9.67	7.15	2.75	1.49	11.97	9.91	2.04	2.13	0.10	0.05
*A. woodii*	Song et al.^44^	9.24	6.71	2.97	1.94	11.39	9.39	2.25	1.98	0.10	0.05
*E. limosum*	Song et al.^46^	7.78	5.44	1.14	0.87	7.14	7.20	0.97	0.06	0.50	0.20
*E. limosum*	Song et al.^44^	6.11	5.86	0.60	0.88	7.13	7.20	0.96	0.06	0.20	0.20
*C. drakei*	Song et al.^44^	12.84	10.01	0.28	1.72	9.82	7.21	0.41	0.60	0.05	0.05
*C. ljungdahlii*	Al-Bassam et al.^24^	10.47	7.91	0.64	1.23	10.52	7.58	1.07	0.51	0.50	0.50

WLP methyl branch											
*A. woodii*	Shin et al.^45^	11.33	8.71	0.57	1.10	13.74	13.03	0.93	1.20	1.00e^-03^	1.10e^-03^
*A. woodii*	Song et al.^44^	9.34	7.08	2.86	1.14	11.73	10.57	1.91	1.11	0.05	0.03
*E. limosum*	Song et al.^46^	11.22	11.14	1.69	1.62	14.32	12.63	1.12	1,19	3.97e^-03^	0.05
*E. limosum*	Song et al.^44^	12.13	11.56	0.94	1.61	14.31	12.63	1.12	1.18	0.02	0.08
*C. drakei*	Song et al.^44^	14.20	11.83	0.12	0.30	9.43	8.43	1.88	0.67	3.97e^-03^	3.97e^-03^
*C. ljungdahlii*	Al-Bassam et al.^24^	12.59	11.77	0.38	0.37	11.45	11.81	0.85	1.44	0.03	0.42

Rnf complex											
*A. woodii*	Shin et al.^45^	9.83	8.73	0.45	0.27	11.66	11.68	0.81	0.52	1.00e^-03^	1.00e^-03^
*A. woodii*	Song et al.^44^	9.38	8.43	0.57	0.42	11.22	11.26	0.83	0.68	1.00e^-03^	1.00e^-03^
*E. limosum*	Song et al.^46^	10.34	7.47	1.34	0.91	11.58	10.86	1.03	0.51	0.05	1.00e^-03^
*E. limosum*	Song et al.^44^	8.25	7.89	0.98	0.92	11.58	10.87	1.03	0.51	1.00e^-03^	4.00e^-03^
*C. drakei*	Song et al.^44^	11.17	8.15	1.42	1.32	3.79	7.31	0.60	1.20	2.00e^-03^	0.15
*C. ljungdahlii*	Al-Bassam et al.^24^	11.63	9.83	0.18	0.63	11.99	10.16	0.14	0.40	0.15	0.41

ATP synthase complex											
*A. woodii*	Shin et al.^45^	10.64	8.39	1.42	0.81	11.40	11.77	1.03	1.19	0.03	1.42e^-06^
*A. woodii*	Song et al.^44^	9.99	7.88	1.90	0.75	11.06	11.23	1.38	1.62	0.05	2.16e^-05^
*E. limosum*	Song et al.^46^	11.14	7.95	2.33	1.74	12.30	11.63	1.27	1.40	0.07	3.47e^-05^
*E. limosum*	Song et al.^44^	10.26	8.37	1.67	1.72	12.29	11.63	1.27	1.40	3.70e^-04^	5.35e^-05^
*C. drakei*	Song et al.^44^	12.93	11.76	2.02	2.45	4.27	9.16	2.08	1.45	1.50e^-04^	0.04
*C. ljungdahlii*	Al-Bassam et al.^24^	10.36	12.33	0.30	0.37	8.31	9.05	1.54	0.77	7.77e^-05^	7.77e^-05^

Bifurcating hydrogenase											
*A. woodii*	Shin et al.^45^	10.38	9.11	2.31	1.04	12.5	11.03	0.53	0.31	0.03	4.00e^-03^
*A. woodii*	Song et al.^44^	9.94	8.76	2.73	1.10	12.12	10.87	0.68	0.84	0.05	4.00e^-03^
*E. limosum*	Song et al.^46^	10.93	8.83	2.26	1.50	13.62	10.67	0.63	1.02	4.00e^-03^	0.02
*E. limosum*	Song et al.^44^	9.50	9.25	1.14	1.51	13.78	10.86	0.88	1.20	4.00e^-03^	0.05
*C. drakei*	Song et al.^44^	13.29	6.24	1.91	3.08	7.18	3.17	1.10	1.15	1.00e^-03^	0.03
*C. ljungdahlii*	Al-Bassam et al.^24^	10.66	9.62	0.36	0.57	11.87	10.96	0.46	0.65	0.06	0.06

Lactate oxidation											
*A. woodii*	Shin et al.^45^	7.86	8.02	0.65	0.90	13.77	8.43	0.94	1.38	1.00e^-03^	0.19
*A. woodii*	Song et al.^44^	7.41	7.78	0.80	1.03	13.28	7.89	0.99	1.,38	1.00e^-03^	0.29
*E. limosum*	Song et al.^46^	5.57	4.43	1.62	1.48	10.30	8.94	0.10	0.47	3.00e^-03^	0.13
*E. limosum*	Song et al.^44^	6.86	4.86	3.20	1.50	10.29	8.94	0.98	0.47	0.08	0.13
*C. drakei*	Song et al.^44^	10.27	3.96	1.05	1.96	4.65	2.92	1.79	1.54	2.00e^-04^	0.06
*C. ljungdahlii*	Al-Bassam et al.^24^	4.37	2.41	2.33	1.58	4.06	2.19	2.19	2.53	0.27	0.15

2,3-BDO metabolism											
*A. woodii*	Shin et al.^45^	6.96	8.63	0.10	0.23	9.84	8.83	0.35	0.34	0.048	0.35
*A. woodii*	Song et al.^44^	6.82	8.48	0.27	0.24	9.37	8.21	0.34	0.32	0.047	0.85
*E. limosum*	Song et al.^46^	5.81	5.70	3.76	3.12	8.19	7.41	1.81	1.95	0.15	0.12
*E. limosum*	Song et al.^44^	6.44	6.12	2.09	3.12	8.18	7.42	1.80	1.95	0.066	0.12
*C. drakei*	Song et al.^44^	6.02	8.66	0.35	1.18	5.99	5.17	4.69	1.04	0.50	0.03
*C. ljungdahlii*	Al-Bassam et al.^24^	6.74	7.16	1.47	1.27	6.13	6.68	2.13	1.17	1.00	0.06

EG metabolism											
*A. woodii*	Shin et al.^45^	11.57	11.85	1.93	0.98	14.64	14.13	1.05	1.04	1.27e^-05^	3.47e^-06^
*A. woodii*	Song et al.^44^	11.22	11.44	2.24	0.97	13.97	13.56	1.38	1.27	1.00e^-04^	2.98e^-05^
*C. drakei*	Song et al.^44^	7.17	4.60	2.11	2.22	1.91	3.30	0.25	2.51	0.07	0.10

Table showing the mRNA counts and ribosome-protected fragments in autotrophic and heterotrophic condition in each acetogen for pathways where translational efficiency was found differentially regulated. Shown are the median (MDN) value and the median absolute deviation (MAD) for each pathway in each dataset. The table also displays the Wilcoxon's rank-sum test p value carried out to assess the statistical significance of the difference between RNA and RPF in each growth condition for each pathway. Abbreviations: BDO: butanediol; EG: ethylene glycol.

When comparing the change in translational efficiency with the change in total mRNA levels, acetogens showed different behaviors. More precisely, genes encoding the WLP carbonyl branch enzymes were found translationally buffered both in *A. woodii* and in *E. limosum*. Indeed, translational efficiency decreased under the autotrophic growth condition relative to the heterotrophic condition (Figure 6) in *A. woodii* (median(log_2_(TE ratio)) = −3.24 or −3.05 according to the dataset, p value = 2.25e^-10^) and in *E. limosum* (median(log_2_(TE ratio)) = −3.77 or −1.70 according to the dataset, p value = 3.81e^-05^), whereas genes were upregulated at the total mRNA level (supplemental data item, Figures S7 and S8). The observed increase in total mRNA levels reflects the essentiality of these genes for autotrophic growth, and the decrease in translational efficiency is likely to take place to face the limited translational capacity in an energy-restricted condition. In contrast to *A. woodii* and *E. limosum*, translational efficiency was not found to change with growth condition (Figure 6) either in *C. ljungdahlii* (median(log_2_(TE ratio) = −0.15) or in *C. drakei* (median(log_2_(TE ratio)) = 0.73), and gene expression changes were consistently detectable at both levels, suggesting that protein synthesis is mainly governed by mRNA abundances in these acetogens (supplemental data item, Figures S9 and S10).

### Methyl branch

We split the discussion of the translation efficiency of the WLP methyl branch in two portions that concern, respectively, the conversion of CO_2_ to formate, and the conversion of formate to Methyl-CoFeSP.

#### CO_2_ to formate

The first step in the methyl branch is the reduction of CO_2_ to formate by selenocysteine- or non-selenocysteine-containing formate dehydrogenase^62^ (FDH). *A. woodii* employs the hydrogen-dependent CO_2_ reductase (HDCR), an FDH complex linked to hydrogenase, to receive electrons directly from H_2_ and convert CO_2_ to formate.^63^ When comparing the mRNA abundance with the rate of translation as estimated by ribosome footprint density under heterotrophic growth condition, translated mRNA levels increased relative to the total mRNA levels as reflection of high translation efficiency exclusively in *A. woodii*, whereas the opposite was observed in *C. drakei* (Table 3). Under autotrophic growth condition, translation efficiency decreased significantly in *A. woodii* and *E. limosum* (Figure 6) and, hence, translation rates were found to reflect more closely the respective mRNA abundances (Table 3). It is interesting to observe that the ribosomal profile of genes encoding the HDCR complex reflects the relative expected translation rates of its subunits. HDCR receives electrons for CO_2_ reduction either by the hydrogenase subunit HydA2, where hydrogen oxidation takes place, or by reduced ferredoxin. Electrons are then transferred to the active site for CO_2_ reduction in FdhF2 via the electron-transferring subunits HycB2/3. Accordingly, the *fdhF2* (RPF_*fdhF2*_ = 562.6 in autotrophic condition and RPF_*fdhF2*_ = 515.6 in heterotrophic condition) and *hycB2* (RPF_*hycB2*_ = 502.6 in autotrophic condition and RPF_*hycB2*_ = 464.7 in heterotrophic condition) genes were translated at an around 2-fold higher rate compared to the *hydA2* gene (RPF_*hydA2*_ = 259.9 under autotrophic condition and RPF_*hydA2*_ = 158.3 under heterotrophic condition).

Genes encoding the HDCR subunits in *A. woodii* as well as the FDH encoding genes in *C. drakei* and in *C. ljungdahlii* were upregulated at the total and translated mRNA level at similar extent and, hence, feature stable translational efficiency between the autotrophic and heterotrophic growth conditions (p value = 0.02 in *A. woodii*, p value = 0.99 in *C. drakei*, p value = 0.88 in *C. ljungdahlii*). FDH encoding genes in *E. limosum* were translationally buffered since the decrease in translation efficiency (p value = 0.008) opposed the increase in mRNA abundance (Figure 6, supplemental data item).

#### Formate to Methyl-CoFeSP

After the reduction of CO_2_ to formate, the hydrolysis of ATP drives the binding of formyl group to THF. From the formyl-THF, water is split off and the produced methenyl-THF is further reduced via methylene- to methyl-THF. Finally, as mentioned earlier, a methyltransferase transfers the methyl group from methyl-THF via CoFeSP to the CODH/ACS. The reactions yielding methyl-THF from the formyl group involve THF-dependent metalloenzymes: a formyl-THF synthetase (Fhs), a formyl-THF cyclohydrolase (FchA), a methylene-THF dehydrogenase (FoID), and a methylene-THF reducatse (MTHFR). The latter enzyme occurs into different types depending on the acetogenic species. *C. ljungdahlii*, *C. drakei*, and *E. limosum* possess a type II MTHFR system consisting of the MetV-MetF complex whereas *A. woodii* uses a type III MTHFR where MetV-MetF is bound to RnfC2, with a 1:1:1 stoichiometric ratio. MTHFR is another example where stoichiometric protein synthesis is achieved at the translational level. The variability detected in the transcript levels of the genes encoding the MTHFR subunits does not reconcile with the expected stoichiometric ratio that is, instead, achieved thanks to translational control. Indeed, the subunits of the MTHFR system showed similar translation rates coherent with their stoichiometric ratio in the complex (e.g., RPF_*metv*_:RPF_*metF*_:RPF_*rnfC2*_ = 1.00:1.04:1.39 under heterotrophic condition and RPF_*metV*_:RPF_*metF*_:RPF_*rnfC2*_ = 1.00:1.02:1.21 under autotrophic condition) by virtue of different translation efficiencies among the encoding genes (e.g., TE_*metF*_/TE_*rnfC2*_ = 6.19 under autotrophic condition).

When comparing the mRNA abundance with the rate of translation in cells grown under heterotrophic condition, translated mRNA levels increased relative to the total mRNA levels as reflection of high translation efficiency in *A. woodii* while *C. drakei* featured high translational dampening by effect of which translated mRNA levels decreased relative to the total mRNA levels. Under autotrophic condition, translation rates increased relative to mRNA abundances in *A. woodii* and *E. limosum* whereas the opposite occurred in C*. drakei* and *C. ljungdahlii* (Table 3).

Interestingly, when we compared the translational efficiency profiles of the two WLP branches, the *E. limosum* carbonyl branch showed on average a more than 3-fold higher translational efficiency relative to the methyl branch during heterotrophic condition (Wilcoxon's rank-sum exact test, p value = 0.0005, irrespectively of the dataset) whereas the methyl branch showed on average a more than 1.5-fold higher translational efficiency relative to the carbonyl branch (Wilcoxon rank-sum exact test, p value = 0.006 according to the dataset by Song et al.^46^) during autotrophic condition. This observation suggests that the genes encoding the methyl and carbonyl branches of the WLP in *E. limosum* are prone to diversified translational control mechanisms and that these mechanisms depend on growth condition. This behavior was not recapitulated in any other acetogen included in our analysis.

When comparing the changes occurring in translational regulation upon growth condition, the genes encoding the WLP methyl branch enzymes revealed to be translationally buffered in *A. woodii* since translation efficiency significantly decreased (median(log_2_(TE^A^)) = 2.54 or 2.61 depending on the dataset; median(log_2_(TE^H^)) = 4.09 or 3.56 depending on the dataset; p value = 0.0002), irrespectively of the dataset, whereas transcript levels increased in cells grown autotrophically compared to cells grown heterotrophically (median(log_2_(RNA^A^/RNA^H^)) = 2.39 or 2.22 depending on the dataset), as shown in Figure S7. Instead, in *E. limosum*, genes encoding the WLP methyl branch were found regulated exclusively at the translational level since translation efficiency increased under autotrophic condition relative to heterotrophic one (median(log_2_(TE^A^)) = 2.68 or 2.18 depending on the dataset; median(log_2_(TE^H^)) = 1.52 or 1.10 depending on the dataset; p value = 0.0009) despite stable transcript levels (Figures 6 and S8, supplemental data item). Finally, genes do not show any significant change in translational efficiency since translational levels were lower than total mRNA levels at similar extent in the two conditions in *C. drakei* (median(log_2_(TE^H^)) = −3.82; median(log_2_(TE^A^)) = −3.88; p value = 0.42) and in *C. ljungdahlii* (median(log_2_(TE^H^)) = −0.94; median(log_2_(TE^A^)) = −0.92; p value = 0.41), as shown in Figures S9 and S10. Therefore, these results suggest that the intervention of translational mechanisms in the regulation of the WLP is species dependent.

### Energy homeostasis

During lithotrophic growth, in addition to ATP production through substrate-level phosphorylation in the acetate kinase reaction, acetogens produce ATP through the generation of a transmembrane electrochemical gradient, which relies upon either the *Rhodobacter nitrogen* fixation (Rnf) complex or the energy-converting ferredoxin-dependent hydrogenase complex (Ech),^64^ depending upon the species, and which is utilized by an ATP synthase to synthesize ATP.^65^ When comparing the mRNA abundance with the rate of translation, translated mRNA levels increased relative to total mRNA levels in *A. woodii* and *E. limosum,* irrespectively of the growth condition (Table 3; Figure S11). On the contrary, *C. drakei* showed a different behavior under the autotrophic or heterotrophic condition. Indeed, the rates of translation of genes encoding Rnf complex subunits were translationally dampened compared to the respective mRNA abundances, when cells were grown autotrophically, but they were comparable under heterotrophic condition (Figure S11). Analogously to previously shown examples, translation rates of the genes encoding the Rnf complex were consistent with their stoichiometric relationships. For instance, translation rates were found to vary within a 2-fold range in *A. woodii* and *C. jungdahlii* in cells grown both heterotrophically and autotrophically.

Genes encoding the Rnf complex subunits tend to be upregulated in the autotrophic relative to the heterotrophic condition at the total mRNA level in all the acetogens included in our analysis. However, comparing the changes in translational efficiency with the changes in mRNA abundance unveiled species-specific traits of translational control. Indeed, *A. woodii* and *C. drakei* genes were found translationally buffered since translational efficiency decreased (p value = 8.54e^-07^ in *A. woodii* and p value = 0.002 in *C. drakei*) counteracting the increase in mRNA levels (Figures 6, S7, and S9). On the contrary, genes did not feature any significant change in translational efficiency in *E. limosum* and *C. ljungdahlii* since the changes at the total mRNA levels were forwarded at the translated mRNA level.

Genes encoding the ATP synthase subunits showed higher translated mRNA levels compared with the respective mRNA levels in *A. woodii* and *E. limosum* whereas the opposite behavior was observed in *C. ljungdahlii,* under heterotrophic growth condition. When cells were grown autotrophically, mRNA abundances were translationally dampened only in *C. drakei* and *C. ljungdahlii* (Figure S12). When comparing the changes occurring in translational regulation upon growth condition, genes encoding the ATP synthase were translationally buffered in *C. drakei*, *A. woodii*, and *E. limosum* since the decrease in translation efficiency (p value = 4.69e^-08^ in *A. woodii*, p value = 6.68e^-06^ in *E. limosum* according to the dataset by Song et al.,^46^ p value = 8.23e^-05^ in *C. drakei*) opposed the increase in mRNA abundance. In *C. ljungdahlii* these genes were similarly upregulated at the transcriptional and translational level with no significant change in translational efficiency. It is worth noting that *E. limosum* encodes two ATP synthases of which just the ATP synthase encoded by the B2M23_RS17660-B2M23_RS17700 (corresponding to ELIM_c3452- ELIM_c3460) cluster is transcriptionally regulated and translationally buffered whereas the ATP synthase encoded by the B2M23_RS18740-B2M23_RS18780 (corresponding to ELIM_c3666-ELIM_c3674) cluster was not differentially regulated.

#### Hydrogenases

Flavin-based electron-bifurcating hydrogenases exert decisive roles amid all the hydrogenases identified in acetogens. Reduced ferredoxin is the electron donor for the reduction of carbon dioxide to carbon monoxide. Moreover, as mentioned earlier, in *A. woodii* the Rnf complex, which couples the exergonic electron flow from reduced ferredoxin to oxidized nicotinamide adenine dinucleotide (NAD^+^) to establish a transmembrane electrochemical Na^+^ gradient that then drives the synthesis of ATP, is the only ion motive enzyme coupled with the process of acetogenesis, which underlines the importance of reduced ferredoxin for the energy metabolism in *A. woodii*. However, reduction of ferredoxin with hydrogen as reductant is an endergonic process. To this aim, *A. woodii* relies on the multimeric [FeFe]-hydrogenase HydABCD that employs flavin-based electron bifurcation to overcome the energetic barrier to reduce ferredoxin by coupling the reaction to the exergonic reduction of NAD^+^.^66^ Translation rates of the genes Awo_c26970-Awo_c27010 (AWO_RS13895-AWO_RS13915) encoding the HydABCD subunits were consistent with subunits stoichiometric ratio. These genes were not found to feature any significant change in translational efficiency in the autotrophic condition when compared to the heterotrophic condition (Figure 6) since upregulation at the total mRNA levels is paralleled by the upregulation at the translated mRNA level at similar extent. *E. limosum* was found to harbor a similar hydrogenase that was proposed to be encoded by the ELIM_c2347-ELIM_c2351 (B2M23_RS11885- B2M23_RS11905) gene cluster, based on sequence similarity of the corresponding subunits.^47^ The genes encoding the electron-bifurcating hydrogenase in *E. limosum* were found to feature an increase in translational efficiency in the autotrophic vs. the heterotrophic condition since the increase in mRNA abundance is amplified at the translational level.

The electron-bifurcating hydrogenase, initially characterized in *C. autoethanogenum*^62^ and putatively identified in *C. drakei*^27^ and *C. ljungdahlii*,^62^ differs from the *A. woodii* electron-bifurcating hydrogenase. Indeed, this hydrogenase is ferredoxin- and nicotinamide adenine dinucleotide phosphate- (NADP)-dependent and forms a functional complex with FDH, as reflected also by the finding that the genes for the two enzymes cluster together in the genome. The complex catalyzes both the reversible formation of H_2_ from reduced ferredoxin and reduced nicotinamide adenine dinucleotide phosphate (NADPH) in a coupled reaction and the reversible reduction of CO_2_ with H_2_ to formate. Genes encoding the electron-bifurcating hydrogenase in *C. drakei* (B9W14_20060-B9W14_20085 or B9W14_RS20180-B9W14_RS20205) were translationally buffered since their translational efficiency decreases (p value = 8.23e^-05^) whereas their transcript levels increased when comparing the autotrophic to the heterotrophic condition (Figures 6 and S9). In *C. ljungdahlii* the electron-bifurcating encoding genes (CLJU_c07030-CLJU_c07080 or CLJU_RS03460-CLJU_RS03485) did not feature any significant change in translational efficiency since upregulation at the total mRNA levels was paralleled by the upregulation at the translated mRNA level at similar extent.

### Alternative substrates’ utilization pathways

Acetogens can derive electrons from sugars, acids such as lactate,^67^ diols such as 2,3-butanediol^68^ and ethylene glycol,^69^ and alcohols such as ethanol,^70^ and more rarely amino acids such as alanine.^71^ Even though the oxidation of organic substrates is shown to lead to the formation of reducing equivalents, which are usually directed into the WLP, resulting in the reduction of CO_2_ to acetate, it is worth noting that the fermentation of some alternative substrates (e.g., 1,2-propanediol) does not involve WLP.^72^ Genes involved in lactate and, albeit to a lesser extent, ethylene glycol metabolism showed differential translational efficiency between the autotrophic and heterotrophic conditions in *A. woodii*. Lactate oxidation is operated by the lactate dehydrogenase (LDH) encoded by *lctD* in complex with an electron-transferring flavoprotein (Etf) whose small (EtfB) and large (EtfA) subunits are encoded, respectively, by *lctB* and *lctC*.^67^ Genes involved in lactate metabolism showed higher translated mRNA levels compared with the respective mRNA levels in *A. woodii* and *E. limosum* whereas the opposite behavior was observed in *C. drakei,* under autotrophic growth condition. When analyzing the changes in translational regulation between the two growth conditions, these genes were found to be buffered both in *C. drakei* and *A. woodii*. However, the relative direction of change between transcription and translation efficiency differed in the two species. Indeed, *A. woodii* genes featured an increase in translation efficiency (p value = 5.56e^-07^) in contrast to the decrease of their mRNA abundances, whereas *C. drakei* genes showed a decrease in their translation efficiency (p value = 2.38e^-05^) despite the increase of their transcript levels (Figure 6). We gathered no evidence of translational control either in *E. limosum* or in *C. ljungdahlii*.

The 2,3-butanediol fermentation pathway^68^^,^^73^ consists of an acetolactate synthase, an acetolactate decarboxylase, and a 2,3-butanediol dehydrogenase. When analyzing the changes in translational efficiency in relation to the changes at the transcript level, transcriptional and translational readouts appeared diversified among acetogens and among the genes within the pathway. *A. woodii* genes were found to increase their translational efficiency (p value = 0.0004) to counteract the decrease in mRNA abundance. In *E. limosum*, we did not gather evidence of statistically significant changes overall in translational efficiency of the genes involved in the pathway upon growth condition (Figure 6). Nonetheless, we note the large catalytic subunit of a putative acetolactate synthase gene encoded by B2M23_RS20180 was exclusively translationally downregulated whereas the small regulatory subunit proposed to be encoded by B2M23_RS10090 increased at the total and translated mRNA level at similar extent*. C. ljungdahlii* 2,3-butanediol dehydrogenase encoding gene is subject to translational efficiency regulation since the upregulation at the mRNA level is amplified at the translational level whereas acetolactate synthase small subunit and acetolactate decarboxylase were exclusively regulated, respectively, at the translational and transcriptional level. The mRNA abundance of the *C. drakei* genes in the pathway decreased in the autotrophic compared to the heterotrophic condition and did not show evidence of overall translational efficiency regulation except for the 2,3-butanediol dehydrogenase that was downregulated at the total mRNA level and upregulated at the translated mRNA level.

As mentioned earlier, ethylene glycol metabolism featured translational efficiency regulation (p value = 0.001 in *A. woodii*) (Figure 6). Its characterization in *A. woodii* unveiled that propane diol dehydratase (PduCDE) and CoA-dependent propionaldehyde dehydrogenase (PduP) proteins, encoded by the pdu gene cluster, catalyze ethylene glycol dehydration to acetaldehyde and its CoA-dependent oxidation to acetyl-CoA.^69^ Most of the genes involved in ethylene glycol metabolism in *A. woodii* are exclusively translationally regulated with no significant change in mRNA abundance, except for the genes encoding the Ado-B12-dependent diol dehydratase subunits that are similarly upregulated at both levels. On the contrary, *C. drakei* genes are stable at both levels and do not show evidence of significant change in translation efficiency. *E. limosum* and *C. ljungdahlii* do not appear to encode key genes in this pathway.

### Conclusions

This perspective attempted to provide a quantitative assessment of the complex relationship between total and translated mRNA profiles by processing gene expression data previously generated in relation to the response of several acetogens under autotrophic (namely CO_2_/H_2_) and heterotrophic growth conditions. Summarizing the results of the analysis, it is possible to conclude the following.

• Growth-dependent translational efficiency differs among acetogenic bacteria.
• Translational efficiency affords stoichiometric production of protein complex constituents.
• Translational buffering is a widespread feature and concerns key pathways of the acetogenic metabolism.

As discussed earlier, translational efficiency was found to remarkably differ among acetogens, with *C. drakei* and *C. ljungdahlii* showing generally lower translational efficiency values compared to *E. limosum* and *A. woodii.* Notably, key pathways such as the WLP carbonyl and methyl branches, the RNF complex, and the lactate oxidation pathway were unveiled to be translationally induced or dampened according to the acetogen considered, which points at species-specific traits of translational control. There remains a need to gain mechanistic insights on *cis*- and *trans*-regulatory features presumably underlying the observed translational readouts both for biological understanding of gene expression and for the control of translation in diverse applications of acetogens.

The current study confirmed in several acetogens several lines of evidence that hint at the regulation of translation rates as a mechanism to quantitatively control the production of proteins engaged in multimeric complexes in proportion to the structural stoichiometry of complex subunits. Examples discussed here encompass functional and biotechnological interesting enzyme complexes such as the HDCR,^63^ the ATP synthesis complex, and the heterotrimeric NADH-oxidizing methylenetetrahydrofolate reductase.^74^

Furthermore, efficiency in mRNA translation was generally found to change in a growth-dependent manner. It was found to increase under the autotrophic compared to the heterotrophic condition in *A. woodii* and *C. ljungdahlii*, whereas the opposite tendency was observed in *C. drakei* and *E. limosum*, which could be indicative of different translational strategies to address limited resources in energy-limited conditions. When extending our perspective on translational regulation from the global scale to the level of individual genes in relevant functional subsystems, acetogens responding to different nutrient conditions were found to feature mRNA-specific translational regulation.

Interestingly, another distinctive feature is the presence of widespread translational buffering according to which the changes in translation efficiency oppose the changes in mRNA abundance,^56^ with the decrease in translational efficiency counteracting the increase in mRNA abundance or vice versa. Translational buffering could, in principle, be attributed to gene-specific regulatory features as well as to limited protein synthesis capacity of the ribosomal machinery. Even though both scenarios could take place and future studies are needed to understand the possible relative contributions, it is possible to interpret the observed translational buffering in the light of energetic considerations. The energetics of acetogens is worse in autotrophic vs. heterotrophic condition. For instance, in *A. woodii* the ATP yield for the synthesis of acetate from acetyl-CoA is 0.3 mol ATP/mol of acetate with H_2_:CO_2_ as substrate. On the contrary, under heterotrophic growth condition, the conversion of glucose to incompletely oxidized end products such as acetate by *A. woodii* provides 4.3 mol ATP/mol of glucose.^6^ Therefore, autotrophic growth is not competitive with respect to the heterotrophic one. As a matter of fact, slower growth rates are accompanied by decreased protein production capacity. Therefore, it is possible to hypothesize that the observed translational buffering could be due to a lower protein synthesis capacity under the autotrophic growth condition relative to the heterotrophic one. In line with this thinking, ribosomal proteins were noted to be subject to translational efficiency downregulation in the autotrophic vs. heterotrophic condition. Noteworthily, translational buffering involved several key pathways of acetogenic metabolism such as the WLP or the complexes involved in energy homeostasis, albeit at different extent across acetogens. For instance, the Rnf complex was found translationally buffered only in *A. woodii* and *C. drakei*, and the electron-bifurcating hydrogenase only in *C. drakei*. It is useful to remark that even though the analysis of total and translated mRNA profiles provides valuable insights, the observation of translational buffering deserves future investigations since quantifying genome-scale ribosome density by ribosome profiling is not able to assess both the initiation and the elongation rates of a gene.^75^ Therefore, studying the mechanisms underlying incongruencies between translational and transcriptional regulation requires jointly accounting for additional aspects to ribosome occupancy such as the rates of different translation stages^76^ and the potential influence of *cis*- and *trans*-regulatory factors.^77^

The comprehensive assessment of the extent to which the synthesis of proteins is controlled suggested that improving our current understanding of gene expression regulatory processes not only at the transcriptional level but also at the translational level is decisive to fully realize acetogens’ biotechnological potential through rational strain engineering. The past studies here integrated have sought to draw correlative relationships between ORF sequence properties and some *cis*-acting regulatory features such as structural accessibility and the conservation of the Shine-Dalgarno sequence in mRNA 5′ UTRs. The systematic review presented here suggests it is important to develop a detailed understanding of the post-transcriptional regulatory networks that orchestrate the metabolism of acetogenic bacteria. Indeed, one of the key knowledge gaps identified is the role of *trans*-acting post-transcriptional regulatory factors that include both non-coding RNAs and RNA-binding proteins. To date, our knowledge of these factors in acetogens is extremely limited^47^ whereas the identification of both non-coding RNAs and RNA-binding proteins and of their regulatory modules requires further investigation to mechanistically understand, possibly with the aid of quantitative modeling approaches, the translational control evidenced by this study.

### Limitations of the study

The insights into translational control provided by the current study of total and translated mRNA profiles require to be investigated in additional acetogenic microorganisms and growth conditions. Moreover, care should be taken in analyzing total and translated mRNA profiles since quantifying genome-scale ribosome density by ribosome profiling is not able to assess both the initiation and the elongation rates of a gene. Therefore, the findings of the current analysis of total and translated mRNA readouts will benefit from the integration of complementary information on translational dynamics. Another limitation that this kind of investigation has to contend with is the paucity of explanatory arguments for the recorded phenomena. Future endeavors are desirable to mechanistically untangle the complex relationship between transcription and translation and thus to fill a significant knowledge gap in the genotype-to-phenotype relationships, thus opening exploitation prospects in biotechnological applications.

### Methods

#### RNA-seq and Ribo-seq datasets collection

Each study included in the current analysis^24^^,^^44^^,^^45^^,^^46^ obtained biological duplicate samples for total RNA-seq and Ribo-seq analysis from *E. limosum*, *A. woodii*, and *C. drakei* cells grown at mid-exponential phase in DSMZ 135 medium supplemented with glucose (5 g/L) or H_2_:CO_2_ (80:20 at 2 bar) gas mix for heterotrophic or autotrophic condition, respectively. *C. ljungdahlii* was grown on PETC medium (ATCC medium 1754) supplied with fructose (4 mM) or H_2_:CO_2_ (80:20 at 1.8 bar). The transcriptional level of each gene was defined as the number of reads or fragments per kilobase of transcript per million read/fragments mapped (RPKM_RNA_ or FPKM_RNA_). The translation level of each gene was defined as the number of reads per kilobase per million RPFs (RPKM_RPF_). Detailed experimental procedures concerning RNA-seq and ribosome profiling library preparation along with deep-sequencing data analysis are available in the referenced studies. Detailed information on mapped sequence reads, average read length, and genomic coverage achieved for each microorganism are provided in Table S2. Data processing in the original studies ensured the quantified gene expression levels were comparable between samples. Fold changes at the total and translated mRNA levels were retrieved from the included studies. Genes showing a fold change (in log (base2) scale) between autotrophic and heterotrophic condition greater than |1| and a q-value <0.01 were deemed differentially expressed genes. This information was used throughout the discussion of translational regulation of individual pathways between the autotrophic and heterotrophic conditions. To assist the interpretation of translational efficiency data on single-pathway basis, the Wilcoxon rank-sum test was carried out to assess the statistical significance of the difference between total and translated mRNA levels in each growth condition for each pathway.

#### Functional gene set enrichment analysis (GSEA)

Functional gene sets were defined based on the annotations of genes of each microorganism to Gene Ontology (GO release 2023-05-10) categories.^78^ Gene annotations to GO categories were retrieved downloading the relevant NCBI RefSeq^79^ GFF3 files (RefSeq release 217).

For each acetogen, GSEA was applied to the genes previously ranked according to the expression changes shown between the autotrophic and heterotrophic conditions, separately, at the transcriptional and translational levels. This analysis assessed the statistical significance of the association between gene differential expression and gene membership to functional gene sets. The tool employed to this aim was the FGSEA method available in the fgsea^80^ R package (q-value <0.05).

FGSEA was then applied to the genes ranked according to the ratio of translation efficiencies in the autotrophic relative to the heterotrophic growth condition. We retained the functional categories that turned out overrepresented at the top or bottom of pre-ranked genes (q-value <0.05).

#### Detection of differential translation efficiency by biological pathway

We assessed whether translational efficiency was differentially regulated between the autotrophic and heterotrophic condition (autotrophic/heterotrophic) for each selected pathway. The statistical procedure accounted for the fact that factorial models involved two factors, namely growth condition and experimental dataset, in *A. woodii* and *E. limosum*, and a single factor, i.e., growth condition, in *C. drakei* and *C. ljungdahlii*. We carried out the Wilcoxon rank-sum test in *C. drakei* and *C. ljungdalii* and the aligned rank transform (ART) for nonparametric two-factorial data analysis (as per the implementation in the ARTool R package) in *A. woodii* and *E. limosum*.^81^ In each contrast, translational efficiency was deemed to vary at the statistical significance of 0.01.

### Data availability

This article analyzed previous public RNA-seq and Ribo-seq processed data available as supplemental information of the four studies included in the article.^24^^,^^44^^,^^45^^,^^46^

Pathway annotations and their respective RNA-seq and Ribo-seq processed data are assembled for the acetogens included in the article and made available in the supplemental data item of the current article.

## Supplemental information

Document S1. Figures S1‒S12, Tables S1, and S2
