# Supplementary material for: Translational efficiency in gas-fermenting bacteria: Adding a new layer of regulation to gene expression in acetogens
Source: iScience. 2023 Nov 2;26(12):108383. doi: 10.1016/j.isci.2023.108383 (PMC10684804; doi:10.1016/j.isci.2023.108383)
Supplement: Document S1. Figures S1‒S12, Tables S1, and S2 [file mmc1.pdf]

iScience, Volume *26*

Supplemental information

Translational efficiency in gas-fermenting bacteria: Adding a new layer of regulation to gene expression in acetogens

Angela Re

Supplemental Information

Divergent differentially expressed functional gene sets at the transcriptional and translational levels

For each acetogen, genes were ranked according to the expression changes (fold change (FC) in log (base 2) scale) between the autotrophic and heterotrophic conditions at the total mRNA level and translated mRNA level, respectively. Functional gene set analysis was carried out to identify functional gene sets that are differentially expressed when comparing the autotrophic to the heterotrophic growth condition. In the following, differentially expressed functionalities are briefly surveyed by distinguishing whether they were identified exclusively at the total mRNA level, exclusively at the translated mRNA level or at both levels.

Differentially expressed gene sets identified uniquely at the transcriptional level. For instance, genes involved in oxidoreductase activity (*C. drakei, E. limosum* and *A. woodii*) were overrepresented only in transcriptionally up-regulated genes and genes involved in the type II fatty acid multi-stage biosynthetic process were overrepresented only in transcriptionally down-regulated genes in *E. limosum*. Genes carrying out pyrimidine ribonucleotide biosynthetic processes, and glycolysis/gluconogenesis were overrepresented only in transcriptionally down-regulated genes in *C. drakei*. Up-regulation of genes related to flagellum-dependent cell motility (encoding basal-body constituents, hook-asociated proteins and flagellar type III secretion system members) was recorded at the total mRNA level but not retained at the translated mRNA one in *C. ljungdahlii*. Translation-related genes and genes engaged in histidine biosynthetic process were overrepresented in down-regulated genes in *C. ljungdahlii*. In some instances, differentially expressed gene sets at the transcriptional level show opposite tendencies in different acetogens such as the genes engaged in the histidine biosynthetic process or in proton motive-force driven ATP synthesis. Indeed, most F_1F_o ATPase subunits were up-regulated in *A. woodii* and down-regulated in *C. ljungdahlii*.

Differentially expressed gene sets identified uniquely at the translational level. The gene sets functionally enriched uniquely at the translational level were largely strain-specific. In *C. drakei*, differentially expressed functional classes encompassed genes related to the biosynthesis of inosine monophosphate, that plays a central role in intracellular purine metabolism, or genes carrying out S-adenosyl-L-methionine binding, GTPase, and ATP binding activities. As regards *C. ljungdahlii*, functional gene sets that turned out differentially regulated uniquely at the translational level include only genes endowed with transmembrane transport activity, mainly belonging to the Major Facilitator Superfamily (MFS), which were upregulated. This observation is not generalizable to other acetogens included in our survey; for instance, genes carrying out ABC-type transporter activity were overrepresented in translationally down-regulated gens in *E. limosum*.

Differentially expressed gene sets both at the transcriptional and at the translational level. Differentially expressed gene sets common to both levels include genes annotated to phosphotransferase activity (namely sugar kinases) and genes involved in the deoxirbonucleotide catabolic process (deoxyribose-phosphate aldolases), which are up-regulated in *A. woodii*. Another example of functional categories preserved at both levels is uroporphyrinogen decarboxylase activity that we observed both in *A. woodii* and in *E. limosum*. In *E. limosum* this outcome corresponds to the up-regulation of several uroporphyrinogen decarboxylases that are branch point enzymes in the biosynthesis of tetrapyrroles, catalyzing the conversion of uroporphyrinogen to coproporphyrinogen in the multi-step pathway from 5-aminolevulinate to protoheme. Bacteria generally contain a diversity of environmentally regulated heme-containing respiratory cytochromes where the heme has a redox function as a one-electron carrier[S1]. However, cytochromes have been scarcely characterized so far in acetogens[S2] even though they were discovered in some acetogens 50 years ago[S3]. Circumstantial evidence suggests cytochromes may be involved in electron transport chains in addition to the ferredoxin-dependent respiratory enzyme complexes, which are either Rnf or Ech[S4]. Another class of hemoproteins in bacteria serves as gas sensors. Examples of these are nitric oxide sensing proteins[S5]. In this regard, it is worth noting that nitric oxide was reported to affect cell growth and product distribution in *C. carboxidivorans* P7^T [S6], and a shadowy existence of nitric oxide reductase activity is beginning to emerge such as in *M. thermoacetica*[S7]. In *A. woodii*, the genes endowed with uroporphyrinogen decarboxylase activity are annotated as trimethylamine (TMA) methyltransferases of class 1. Acetogens, including *A. woodii*, are known to grow on methyl-group-containing substrates such as proline betaine[S8], carnitine[S9] and methanol[S10]. Even though *A. woodii* harbours 30 methyltransferase systems[S11], they are largely unexplored[S12]. During growth of *E. maltosivorans* on H_2/CO_2 two TMA methyltransferases were found to be highly overproduced compared to growth on glucose although their function(s) remain unknown[S13]. Methanol supply was found to speed up CO_2/H_2 fermentation by *E. limosum* KIST612[S14]. Furthermore, the MttB enzyme is known to catalyze the THF methylation of proline betaine forming the key intermediate methyl-THF in the WLP[S8]. Therefore, the role of TMA

methyltransferases in autotrophic conditions is worth of further investigation. A final note concerns the translation-related genes, encoding mainly 30S and 50S ribosomal proteins, which were consistently found down-regulated, both at the total mRNA and at the translated mRNA level, in *E. limosum*.

Supplemental figures

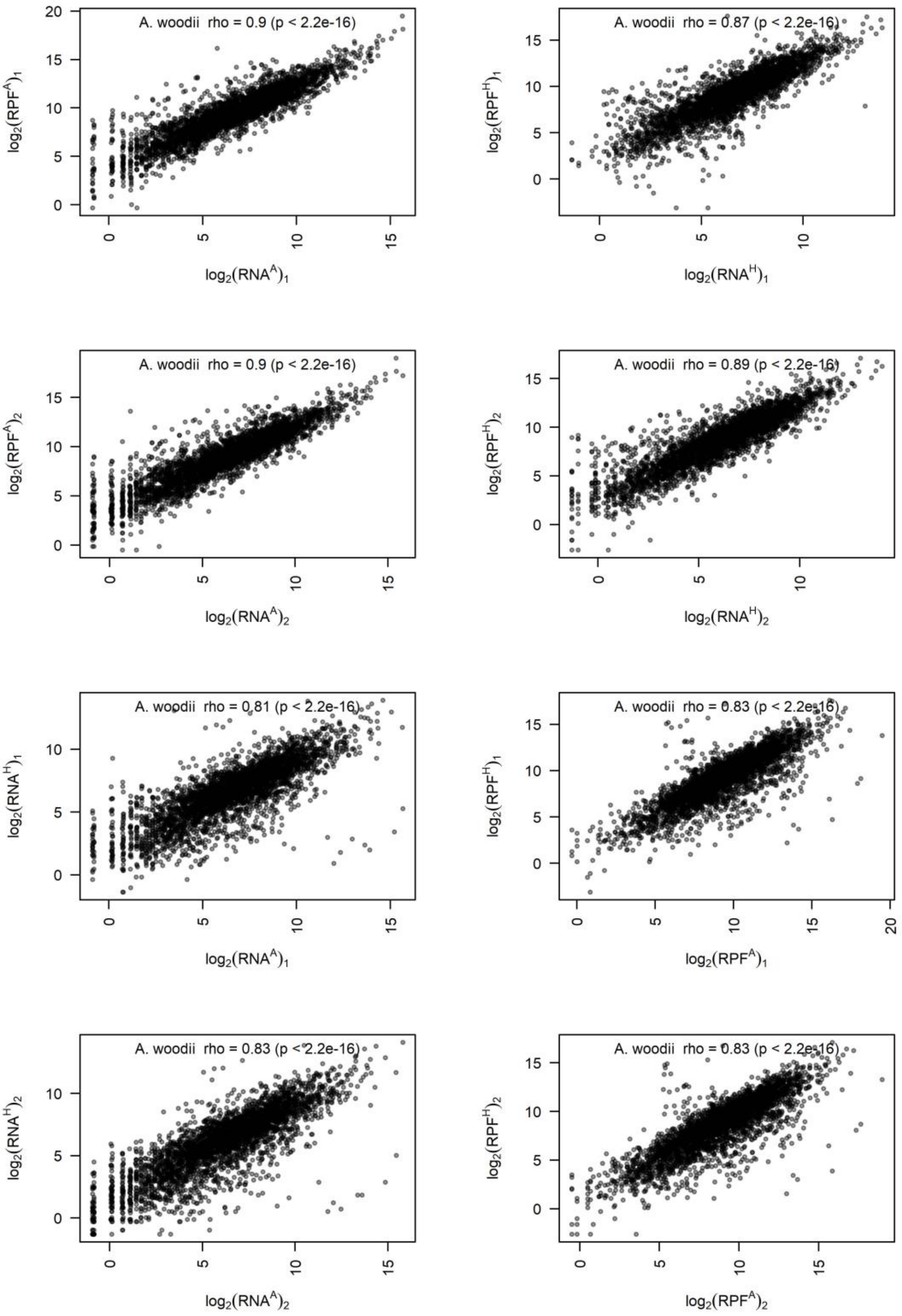

Figure S1. Correlation between gene expression profiles in *A. woodii*, Related to Figure 2. The figure displays the correlation between *A. woodii* total mRNA and translated mRNA levels under the autotrophic or heterotrophic condition and the correlation of *A. woodii* total mRNA profiles or translated mRNA profiles between the two growth conditions.

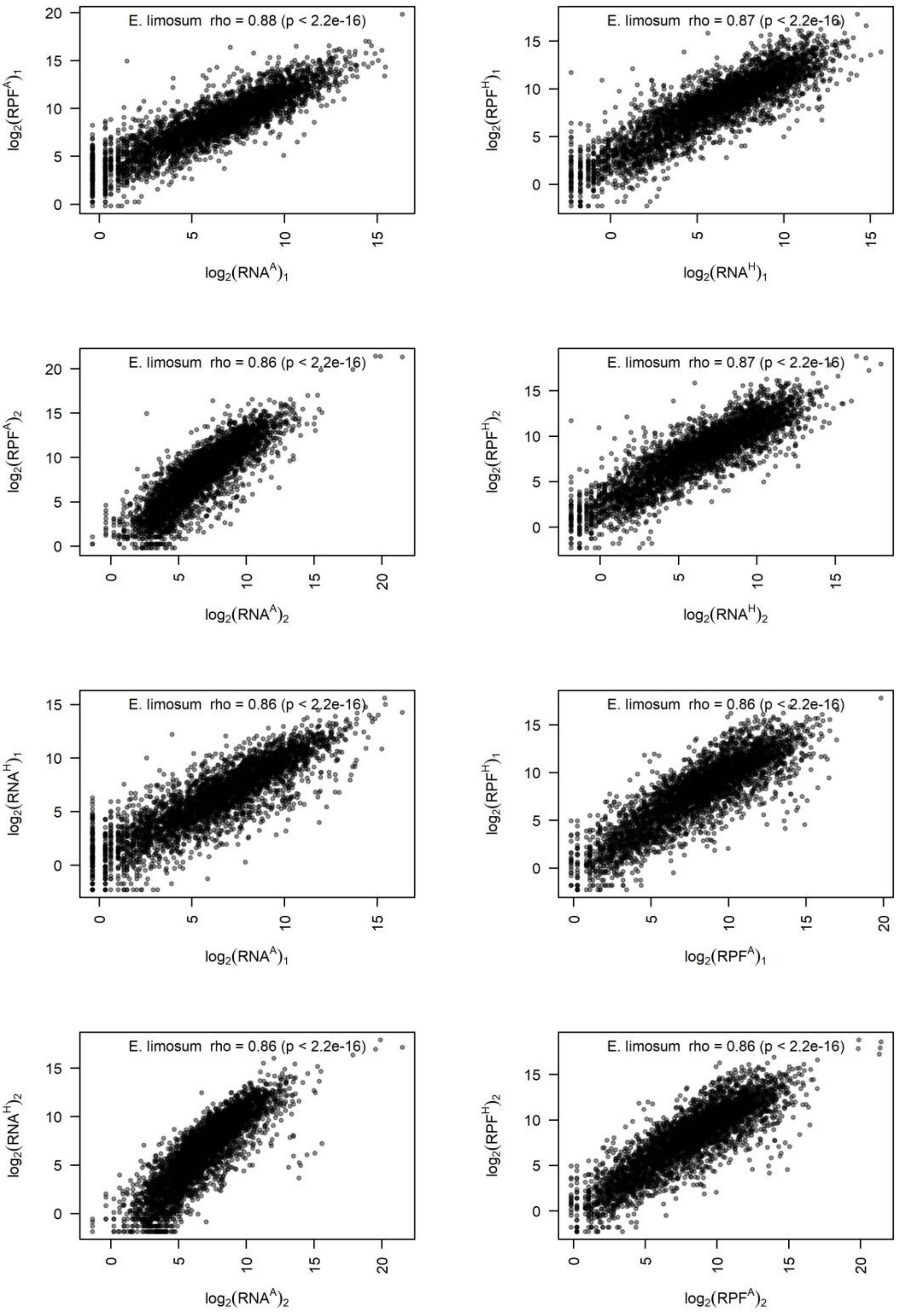

Figure S2. Correlation between gene expression profiles in *E. limosum*, Related to Figure 2*.* The figure displays the correlation between *E. limosum* total mRNA and translated mRNA levels under the autotrophic or heterotrophic condition and the correlation of *E. limosum* total mRNA profiles or translated mRNA profiles between the two growth conditions.

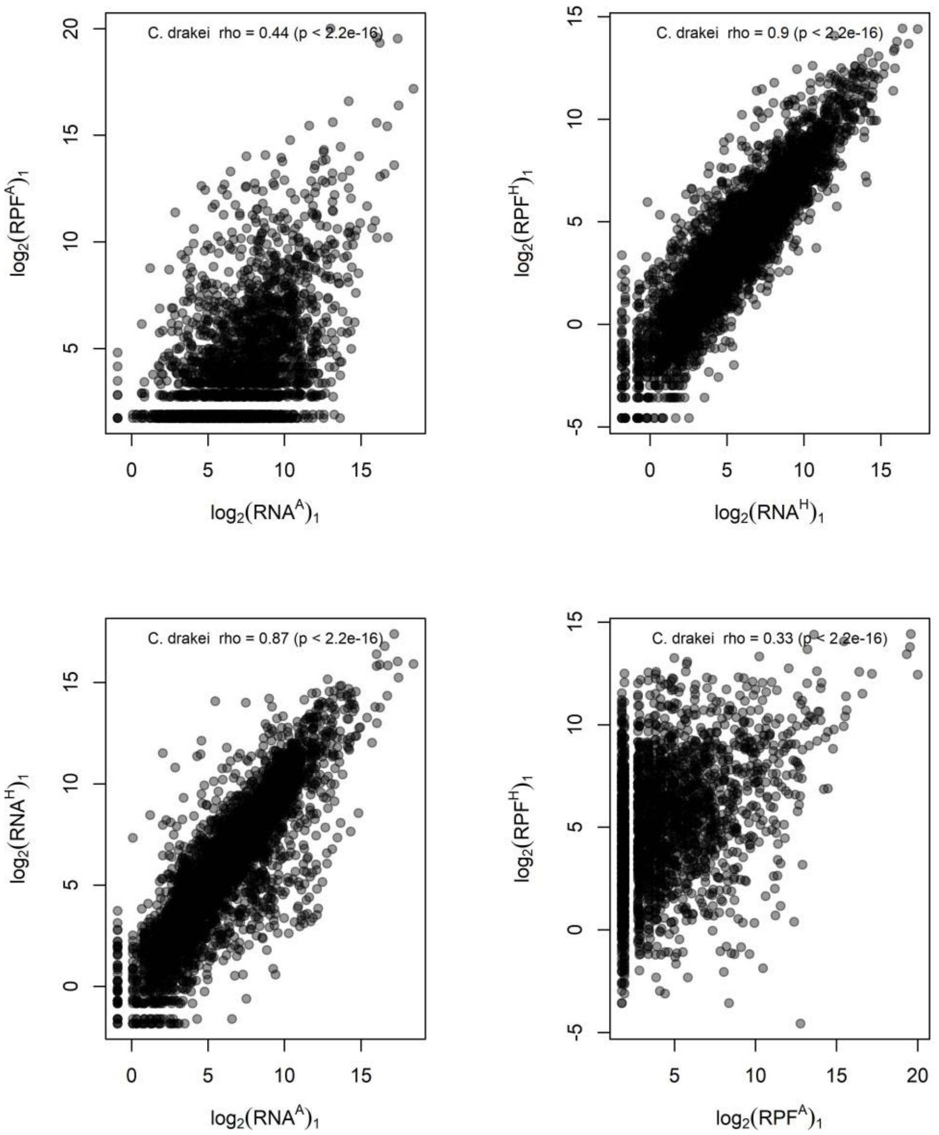

Figure S3. Correlation between gene expression profiles in *C. drakei*, Related to Figure 2. The figure displays the correlation between *C. drakei* total mRNA and translated mRNA levels under the autotrophic or heterotrophic condition and the correlation of *C. drakei* total mRNA profiles or translated mRNA profiles between the two growth conditions.

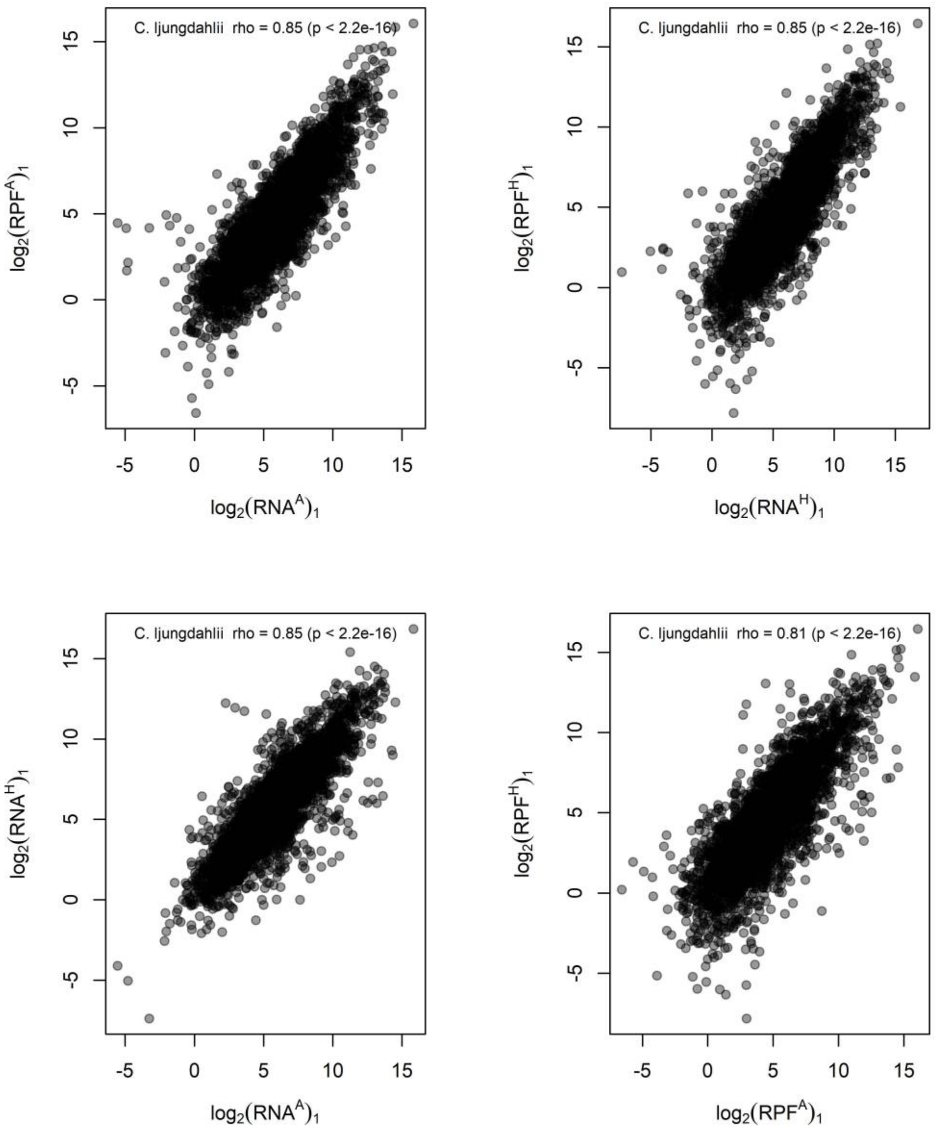

Figure S4. Correlation between gene expression profiles in *C. ljungdahlii*, Related to Figure 2. The figure displays the correlation between *C. ljungdahlii* total mRNA and translated mRNA levels under the autotrophic or heterotrophic condition and the correlation of *C. ljungdahlii* total mRNA profiles or translated mRNA profiles between the two growth conditions.

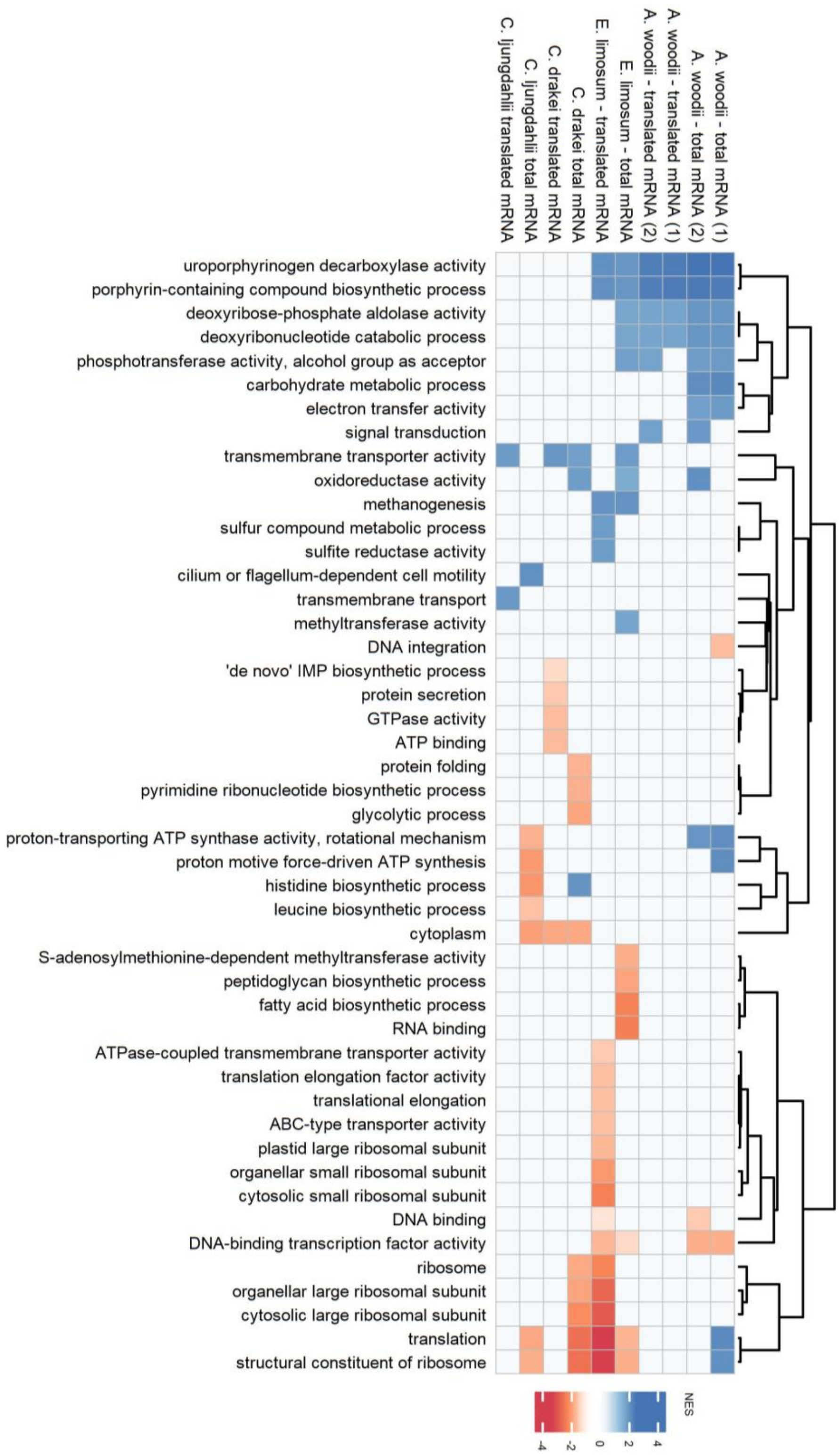

Figure S5. GSEA unveils functionalities affected by translational regulation, Related to Figure 4. Heatmap showing the normalized enrichment score (NES) value corresponding to differentially regulated Gene Ontology (GO) categories in gene ranked according to their translational efficiency ratio. Row names display the acetogen and the data set providing the RNA-Seq and Ribo-seq profiles. Positive (negative) NES values indicate that the genes belonging to a GO category are overrepresented at the top (bottom) of the pre-ranked genes.

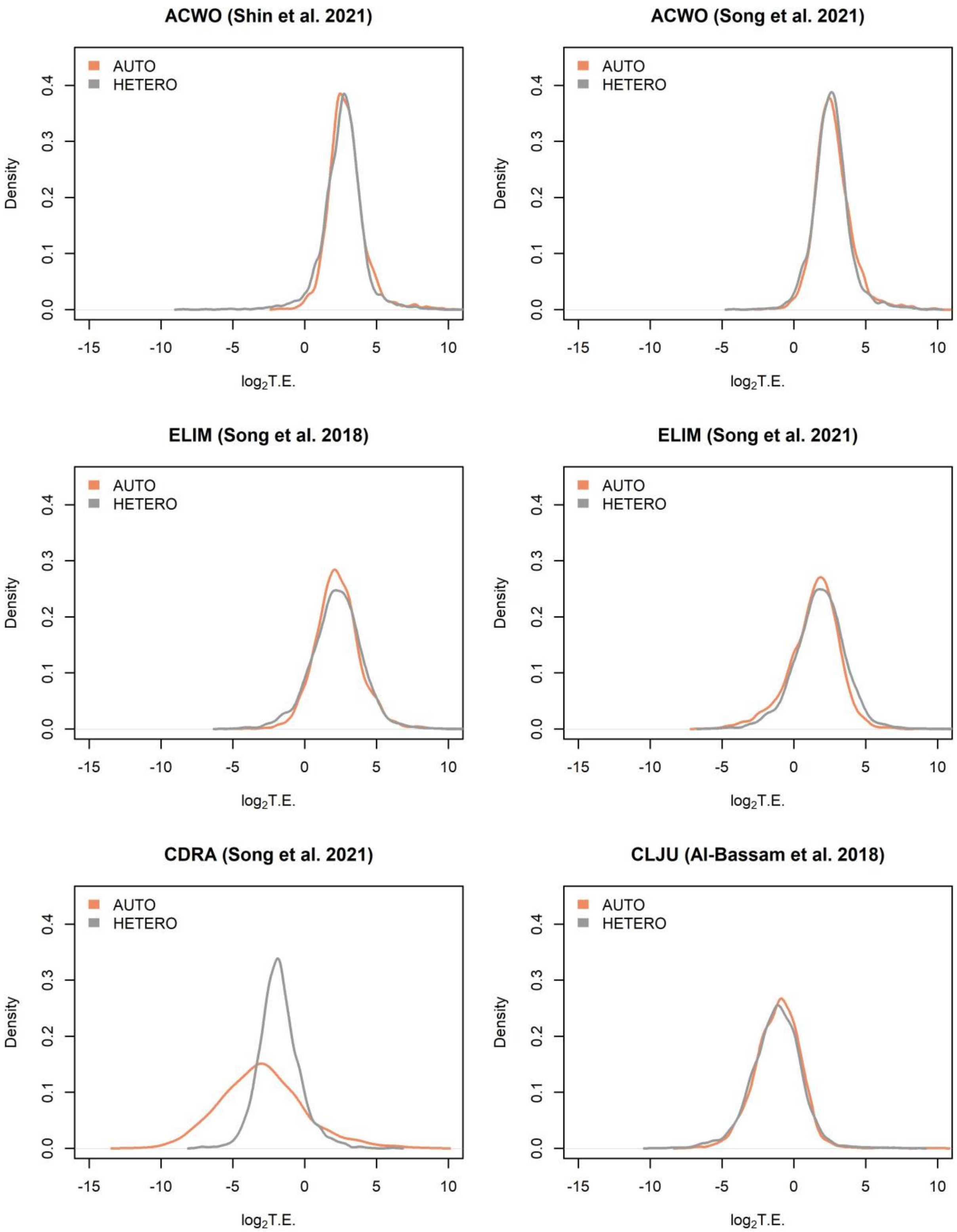

Figure S6. Comparison of translational efficiency between autotrophic and heterotrophic conditions by acetogen, Related to Table 2. Figure panels display the comparison of density plots of translational efficiency (log (base 2) scale) computed for each acetogen under the autotrophic or heterotrophic growth condition.

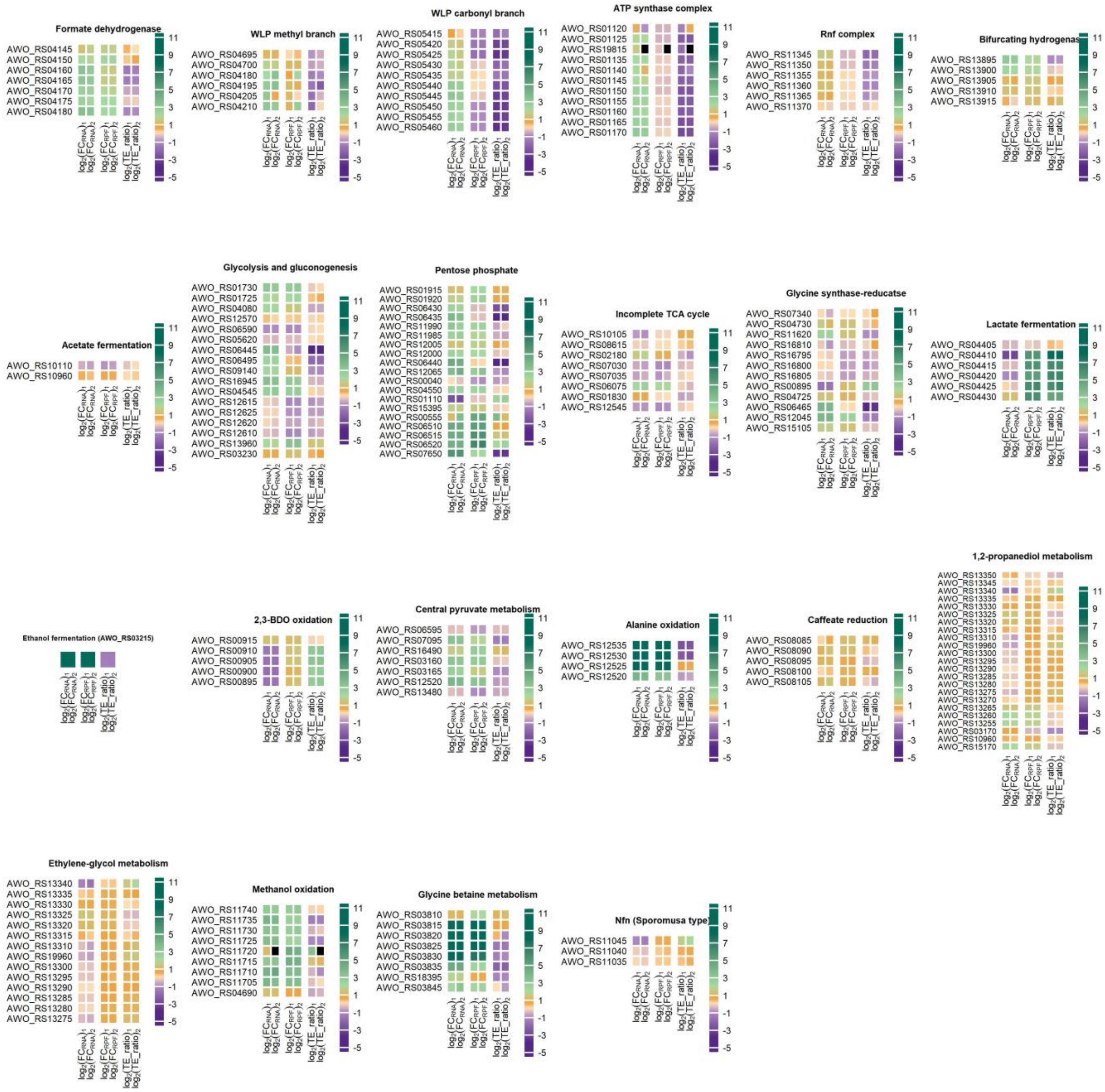

Figure S7. Differential gene expression regulation of major selected pathways of *A. woodii* grown under autotrophic and heterotrophic conditions, Related to Figure 6. Heatmaps showing gene expression changes and translation efficiency ratios (log (base 2) scale) for each gene annotated to major functional modules. Gene expression changes are displayed both at the total mRNA level (herein denoted by “RNA”) and at the translated mRNA level (herein denoted by “RPF”). Row names report NCBI locus tags for the genes encoding the enzymes belonging to a functional module.

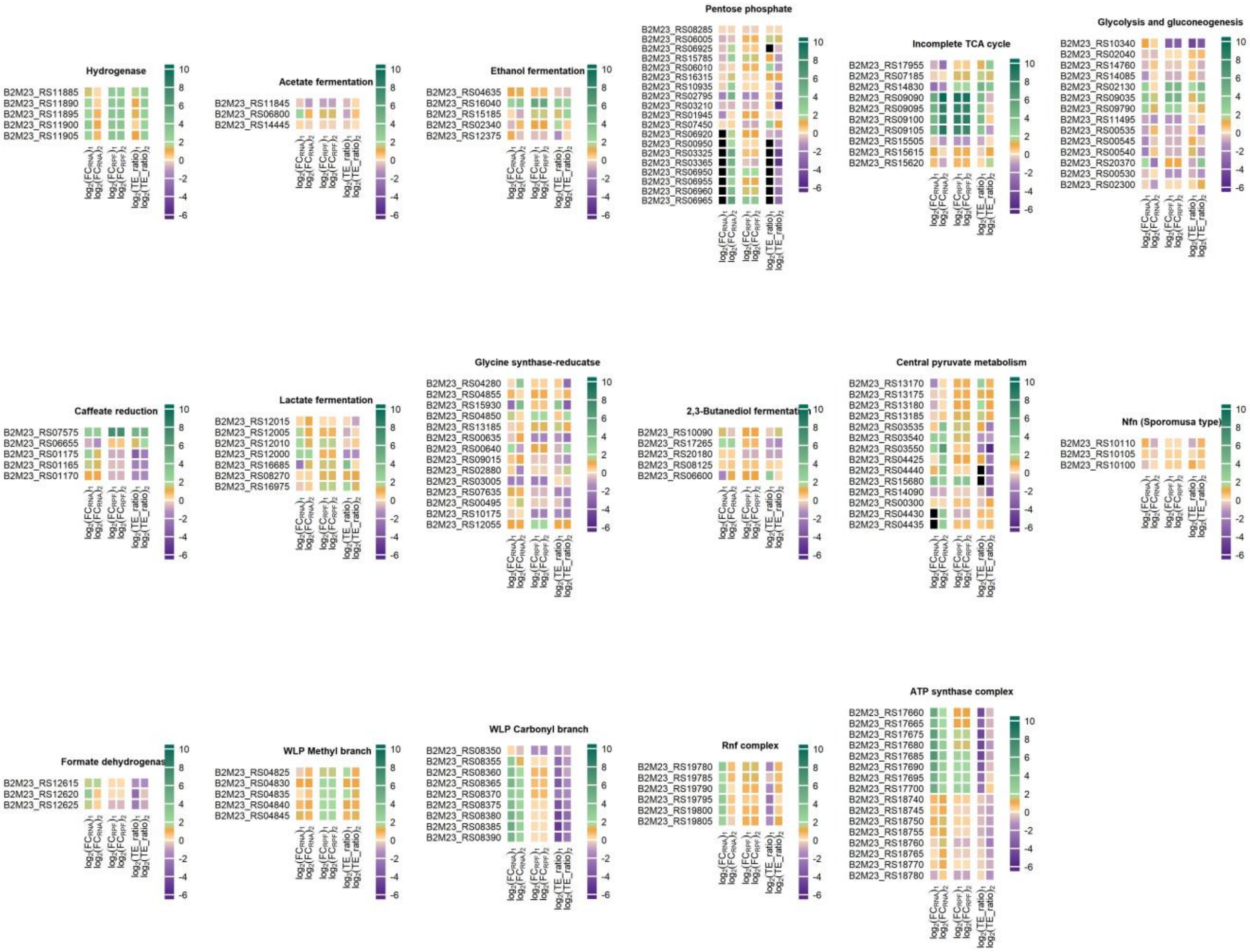

Figure S8. Differential gene expression regulation of major selected pathways of *E. limosum* grown under heterotrophic and autotrophic conditions, Related to Figure 6. Heatmaps showing gene expression changes and translation efficiency ratios (log (base 2) scale) for each gene annotated to major functional modules. Gene expression changes are displayed both at the total mRNA level (herein denoted by “RNA”) and at the translated mRNA level (herein denoted by “RPF”). Row names report NCBI locus tags for the genes encoding the enzymes belonging to a functional module.

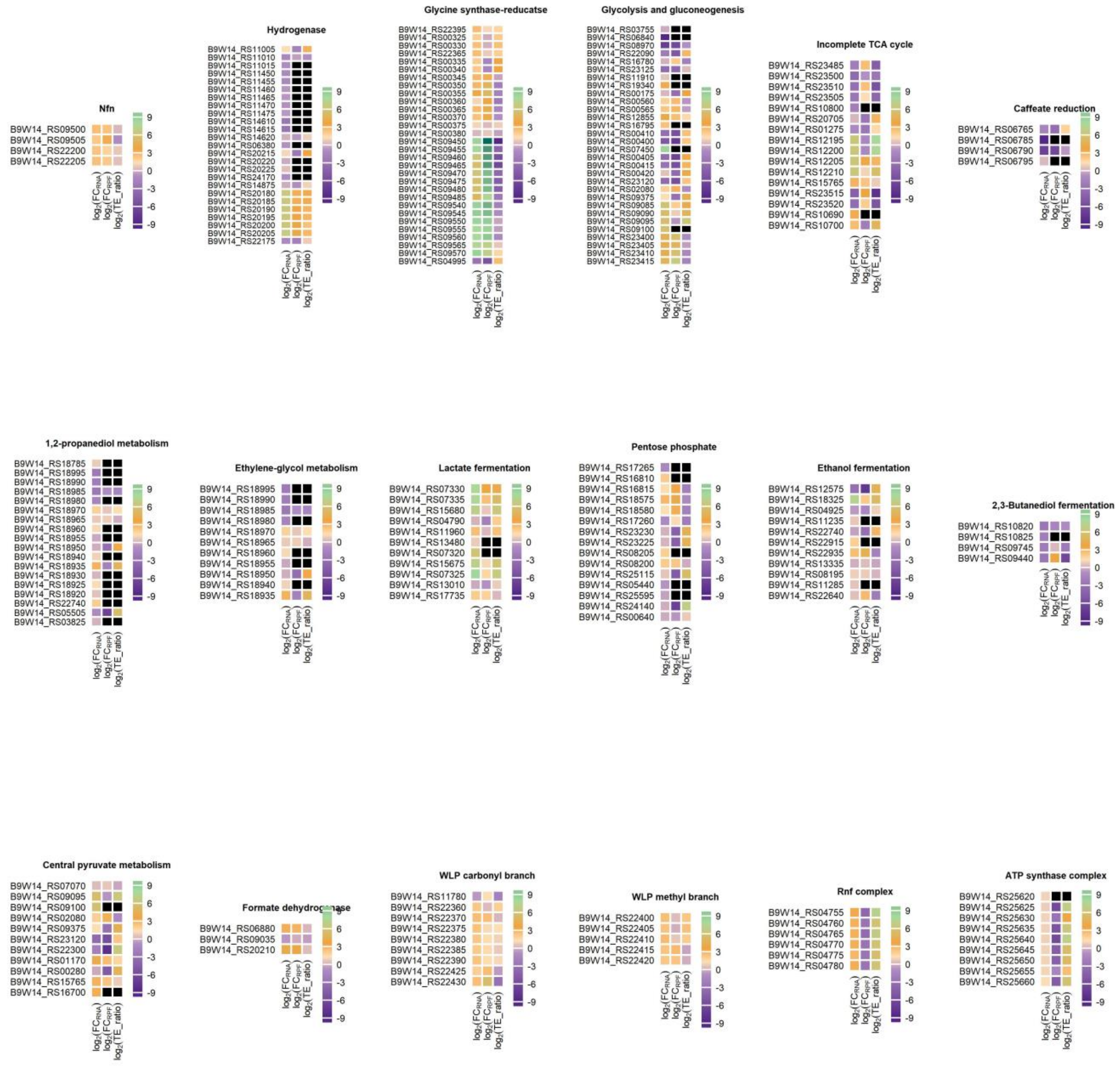

Figure S9. Differential gene expression regulation of major selected pathways of *C. drakei* grown under heterotrophic and autotrophic conditions, Related to Figure 6. Heatmaps showing gene expression changes and translation efficiency ratios (log (base 2) scale) for each gene annotated to major functional modules. Gene expression changes are displayed both at the total mRNA level (herein denoted by “RNA”) and at the translated mRNA level (herein denoted by “RPF”). Row names report NCBI locus tags for the genes encoding the enzymes belonging to a functional module.

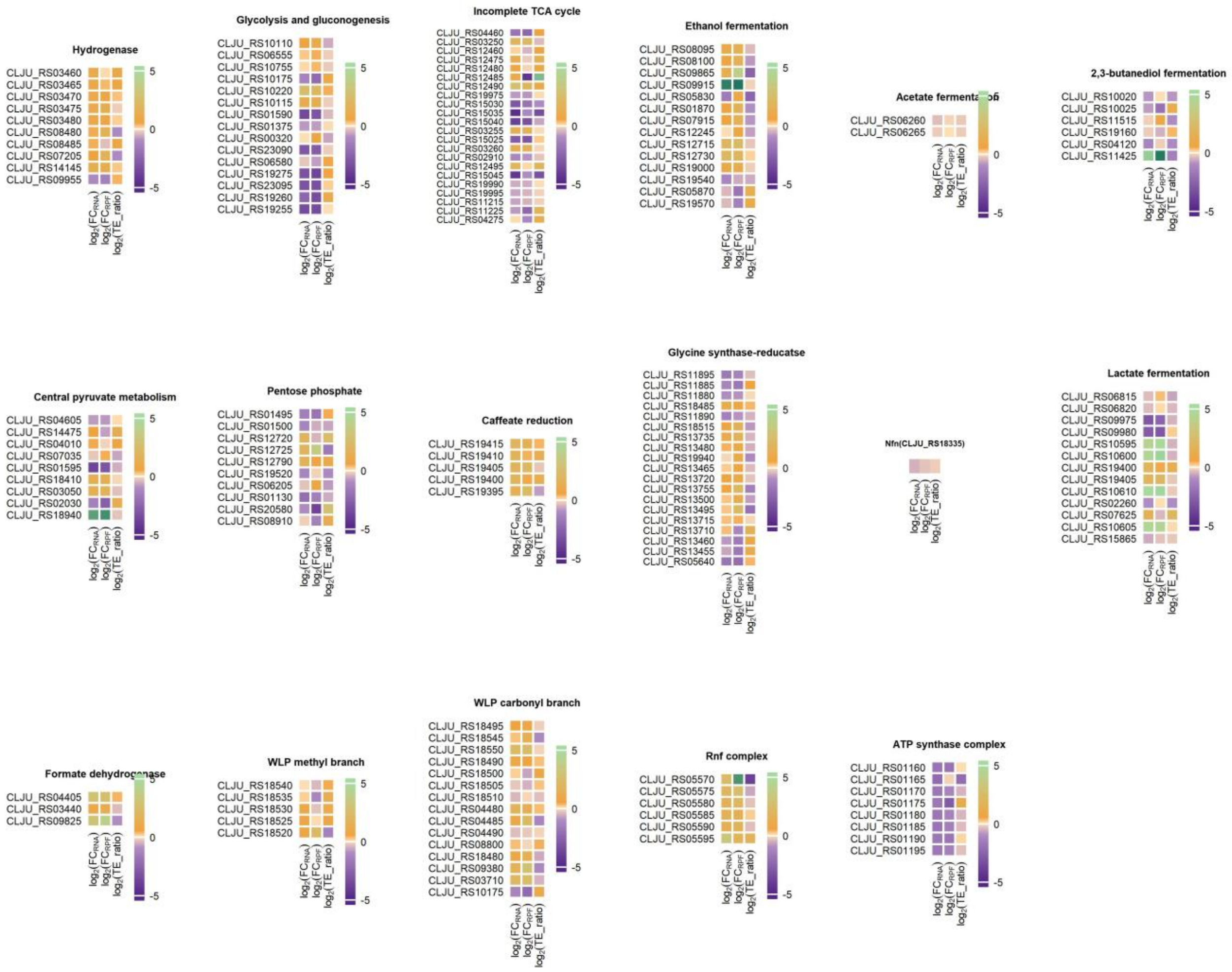

Figure S10. Differential gene expression regulation of major selected pathways of *C. ljungdahlii* grown under heterotrophic and autotrophic conditions, Related to Figure 6. Heatmaps showing gene expression changes and translation efficiency ratios (log (base 2) scale) for each gene annotated to major functional modules. Gene expression changes are displayed both at the total mRNA level (herein denoted by “RNA”) and at the translated mRNA level (herein denoted by “RPF”). Row names report NCBI locus tags for the genes encoding the enzymes belonging to a functional module.

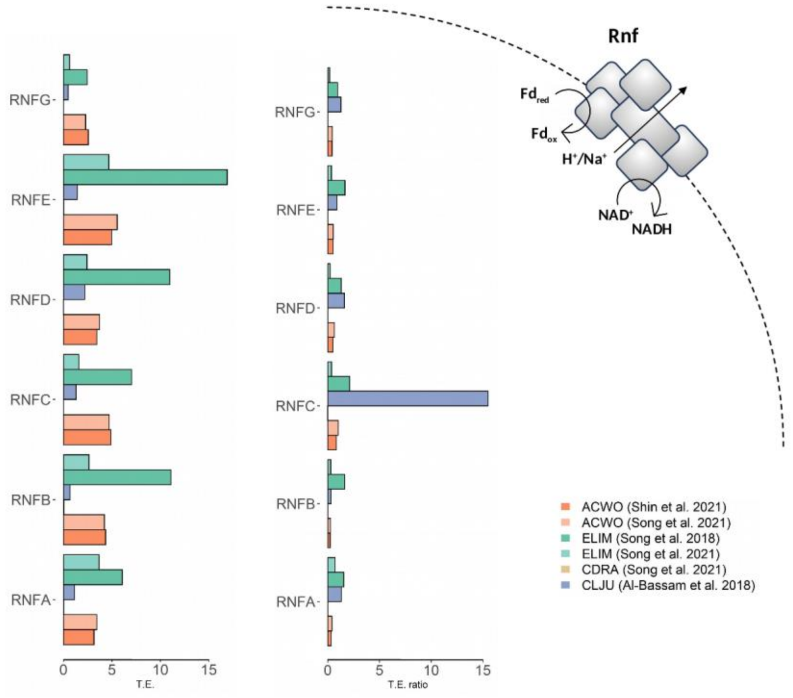

Figure S11. Translational efficiency and translational efficiency ratio of the Rnf complex, Related to Figure 6. For each subunit of the Rnf complex, the figure displays translational efficiency in the autotrophic condition and the ratio of translational efficiency between the autotrophic and heterotrophic conditions in each acetogen (color-coded as per the legend).

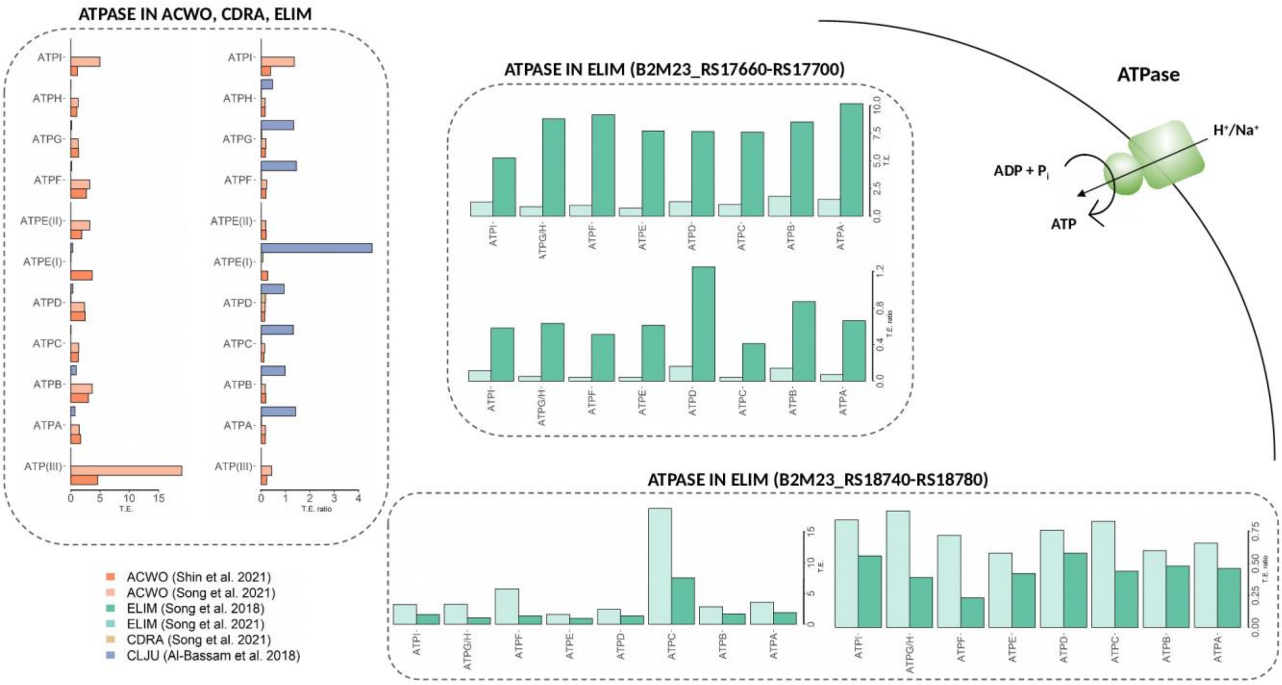

Figure S12. Translational efficiency and translational efficiency ratio of the ATP synthesis complex, Related to Figure 6. For each subunit of the ATP synthesis complex, the figure displays translational efficiency in the autotrophic condition and the ratio of translational efficiency between the autotrophic and heterotrophic conditions in each acetogen (color-coded as per the legend). For *A. woodii*, atpE occurs in three instances, which are indicated by the I, II, and III suffix according to the annotations reported in Supplementary Data. For sake of cleanness, data in *E. limosum* is shown separately from the remaining since the *E. limosum* genome was found to encode two ATPases.

Supplemental tables

Strain	Dataset	Spearman rank correlation
A. woodii	Shin et al.[S17]	0,39
A. woodii	Song et al.[S16]	0,45
E. limosum	Song et al.[S18]	0,22
E. limosum	Song et al.[S16]	0,20
C. ljungdahlii	Al-Bassam et al.[S15]	0,71
C. drakei	Song et al.[S16]	0,24

Table S1. Comparison between translation efficiency values in the autotrophic and heterotrophic conditions, Related to Figure 2. Displayed is the Spearman rank correlation between translation efficiency values in the autotrophic and heterotrophic conditions in each acetogen.

Microorganism	Feature	RNA-seq	Ribo-seq	Reference
Eubacterium limosum ATCC 8486	mapped sequence reads	19,8 x 10^6	255 x 10^6	Song et al.[S18]
Eubacterium limosum ATCC 8487	average read length (nt)	130 bp	32 bp	Song et al.[S18]
Eubacterium limosum ATCC 8488	genomic coverage (x)	146,1	N/A	Song et al.[S18]
Acetobacterium woodii DSM 1030	mapped sequence reads	1,2-4,7 x 10^6	10,3 x 10^6	Shin et al.[S17]
Acetobacterium woodii DSM 1030	average read length (nt)	142-149 bp	30-32 bp	Shin et al.[S17]
Acetobacterium woodii DSM 1030	genomic coverage (x)	42.8	87.8	Shin et al.[S17]
Acetobacterium woodii DSM 1030	mapped sequence reads	1,2-4,7 x 10^6	16,4-57,7 x 10^6	Song et al.[S16]
Acetobacterium woodii DSM 1030	average read length (nt)	142,4-149,9 bp	33,9-37,4	Song et al.[S16]
Acetobacterium woodii DSM 1030	genomic coverage (x)	42.8-174.5	137.2-490.9	Song et al.[S16]
Clostridium drakei DSM 12750	mapped sequence reads	2,2-6,9 x 10^6	2,0-16,6 x 10^6	Song et al.[S16]
Clostridium drakei DSM 12750	average read length (nt)	139-142 bp	35,4-35,9	Song et al.[S16]
Clostridium drakei DSM 12750	genomic coverage (x)	54-173	12.4-104.2	Song et al.[S16]
Eubacterium limosum DSM 20543	mapped sequence reads	5,4-8,3 x 10^6	14,4-29,7 x 10^6	Song et al.[S16]
Eubacterium limosum DSM 20543	average read length (nt)	135,6-138,7 bp	32,2-34,5	Song et al.[S16]
Eubacterium limosum DSM 20543	genomic coverage (x)	169,0-259,8	112,3-216,2	Song et al.[S16]
Clostridium ljungdahlii ATCC 55383	mapped sequence reads	35,7-140,6 x 10^6	1,6-7,2 x 10^6	Al-Bassam et al.[S15]
Clostridium ljungdahlii ATCC 55383	average read length (nt)	N/A	N/A	Al-Bassam et al.[S15]
Clostridium ljungdahlii ATCC 55383	genomic coverage (x)	2212-3710	N/A	Al-Bassam et al.[S15]

Table S2. Summary statistics of RNA-Seq and Ribo-Seq data, Related to Methods. Datasets were acquired for *E. limosum*, *A. woodii*, *C. ljungdahlii* and *C. drakei*. N/A indicates that the study does not provide the information.

Supplemental Data Item. **Gene annotations at single pathway level, Related to Figure 6.** The file is organized in four sheets corresponding to the four acetogens included in our survey. Each sheet describes the genes annotated to each of the major pathways selected for closer examination by providing details pertaining their genomic location and gene/protein identifiers as well as their gene expression data at the total mRNA and translated mRNA level, as they are extracted from the studies included in our survey. Each pathway is associated with a “Processig” status where the logic value “YES” (“NO”) indicates that genes known to be essential to make a pathway functional are (not) identifiable in the genome of the acetogen of interest.

Supplemental references

1. Dailey, H.A., Dailey, T.A., Gerdes, S., Jahn, D., Jahn, M., O'Brian, M.R., and Warren, M.J. (2017). Prokaryotic heme biosynthesis: multiple pathways to a common essential product. Microbiol Mol Biol Rev. 81, e00048-16. 10.1128/MMBR.00048-16.
2. Di Leonardo, P.F., Antonicelli, G., Agostino, V., and Re, A. (2022). Genome-scale mining of acetogens of the genus *Clostridium* unveils distinctive traits in [FeFe]- and [NiFe]-hydrogenase content and maturation. Microbiol Spectr. 10, e0101922. 10.1128/spectrum.01019-22.
3. Rosenbaum, F.P., and Müller, V. (2021). Energy conservation under extreme energy limitation: the role of cytochromes and quinones in acetogenic bacteria. Extremophiles 25, 413-424. 10.1007/s00792-021-01241-0.
4. Katsyv, A., and Müller V. (2020). Overcoming energetic barriers in acetogenic C_1 Conversion. Front Bioeng Biotechnol. 8, 621166. 10.3389/fbioe.2020.621166.
5. Plate, L., and Marletta, M.A. (2013). Nitric oxide-sensing H-NOX proteins govern bacterial communal behavior. Trends Biochem Sci. 38, 566-75. 10.1016/j.tibs.2013.08.008.

6. Ahmed, A., and Lewis, RS. (2007). Fermentation of biomass-generated synthesis gas: effects of nitric oxide. Biotechnol Bioeng. 97, 1080-6. 10.1002/bit.21305.
7. Silaghi-Dumitrescu, R., Coulter, E.D., Das, A., Ljungdahl, L.G., Jameson, G.N., Huynh, B.H., Kurtz, and D.M. Jr. (2003). A flavodiiron protein and high molecular weight rubredoxin from *Moorella thermoacetica* with nitric oxide reductase activity. Biochemistry 42, 2806-15. 10.1021/bi027253k.
8. Picking, J.W., Behrman, E.J., Zhang, L., and Krzycki, J.A. (2019). MtpB, a member of the MttB superfamily from the human intestinal acetogen *Eubacterium limosum*, catalyzes proline betaine demethylation. J Biol Chem. 294, 13697-13707. 10.1074/jbc.RA119.009886.
9. Kountz, D.J., Behrman, E.J., Zhang, L., and Krzycki, J.A. (2020). MtcB, a member of the MttB superfamily from the human gut acetogen *Eubacterium limosum*, is a cobalamin-dependent carnitine demethylase. J Biol Chem. 295, 11971-11981. 10.1074/jbc.RA120.012934.
10. Kremp, F., Poehlein, A., Daniel, R., and Müller, V. (2018). Methanol metabolism in the acetogenic bacterium *Acetobacterium woodii*. Environ Microbiol. 20, 4369-4384. 10.1111/1462-2920.14356.
11. Poehlein, A., Schmidt, S., Kaster, A.K., Goenrich, M., Vollmers, J., Thürmer, A., Bertsch, J., Schuchmann, K., Voigt, B., Hecker, M., et al. (2012). An ancient pathway combining carbon dioxide fixation with the generation and utilization of a sodium ion gradient for ATP synthesis. PLoS One 7, e33439. 10.1371/journal.pone.0033439.
12. Kremp, F., and Müller, V. (2021). Methanol and methyl group conversion in acetogenic bacteria: biochemistry, physiology and application. FEMS Microbiol Rev. 45, fuaa040. 10.1093/femsre/fuaa040.
13. Feng, Y., Bui, T.P.N., Stams, A.J.M., Boeren, S., Sánchez-Andrea, I., de Vos, W.M. (2022). Comparative genomics and proteomics of *Eubacterium maltosivorans*: functional identification of trimethylamine methyltransferases and bacterial microcompartments in a human intestinal bacterium with a versatile lifestyle. Environ Microbiol. 24, 517-534. 10.1111/1462-2920.15886.
14. Kim, J.Y., Park, S., Jeong, J., Lee, M., Kang, B., Jang, S.H., Jeon, J., Jang, N., Oh, S., Park, Z.Y., et al. (2021). Methanol supply speeds up synthesis gas fermentation by methylotrophic-acetogenic bacterium, *Eubacterium limosum* KIST612. Bioresour Technol. 321, 124521. 10.1016/j.biortech.2020.124521.
15. Al-Bassam, M.M., Kim, J.N., Zaramela, L.S., Kellman, B.P., Zuniga, C., Wozniak, J.M., Gonzalez, D.J., and Zengler, K. (2018). Optimization of carbon and energy utilization through differential translational efficiency. Nat Commun. 9, 4474. 10.1038/s41467-018-06993-6.
16. Song, Y., Bae, J., Shin, J., Jin, S., Lee, J.K., Kim, S.C., Cho, S., and Cho, B-K. (2021). Transcriptome and translatome of CO_2 fixing acetogens under heterotrophic and autotrophic conditions. Sci Data 8, 51. 10.1038/s41597-021-00837-7.
17. Shin, J., Song, Y., Kang, S., Jin, S., Lee, J.K., Kim, D.R., Cho, S., Müller, V., and Cho, B-K. (2021). Genome-scale analysis of *Acetobacterium woodii* identifies translational regulation of acetogenesis. mSystems 6, e0069621. 10.1128/mSystems.00696-21.
18. Song, Y., Shin, J., Jin, S., Lee, J.K., Kim, D.R., Kim, S.C., Cho, S., and Cho, B-K. (2018). Genome-scale analysis of syngas fermenting acetogenic bacteria reveals the translational regulation for its autotrophic growth. BMC Genomics. 19, 837. 10.1186/s12864-018-5238-0.
